# Inequalities in the health, nutrition, and wellbeing of Afrodescendant women and children: A cross-sectional analysis of ten Latin American and Caribbean countries

**DOI:** 10.1016/j.lana.2022.100345

**Published:** 2022-08-19

**Authors:** Janaína Calu Costa, Oscar J. Mujica, Giovanna Gatica-Domínguez, Sandra del Pino, Liliana Carvajal, Antonio Sanhueza, Sonja Caffe, Cesar G. Victora, Aluísio J.D. Barros

**Affiliations:** a International Center for Equity in Health, Federal University of Pelotas, Brazil; b Pan American Health Organization, Washington D.C., USA; c Division of Data, Analytics, Planning and Monitoring, Data and Analytics Section, UNICEF, New York, USA; d Department of Global Public Health, Karolinska Institutet, Stockholm, Sweden

**Keywords:** Ethnic and racial disparities, Health equity, Child health, Maternal health, Latin America and the Caribbean

## Abstract

**Background:**

Afrodescendants are systematically affected by discrimination in the Americas and few multi-country studies addressed ethnic inequalities in health and wellbeing in the region. We aimed to investigate gaps in coverage of key health outcomes and socioeconomic inequalities between Afrodescendants and non-Afrodescendants populations in Latin American and Caribbean countries.

**Methods:**

Using national household surveys (2011–2019) from ten countries, we analyzed absolute inequalities between Afrodescendants and a comparison group that includes non-Afrodescendants and non-Indigenous individuals (henceforth non-Afrodescendants) across 17 indicators in the continuum of reproductive, maternal, newborn, child, and adolescent health. These include indicators of family planning, antenatal care, delivery assistance, child nutrition, immunization coverage, child protection, access to improved water, sanitation and hygiene, adolescent fertility, and early childhood mortality. Inequalities between country-specific subgroups of Afrodescendants were also explored. The slope index of inequality was used to assess wealth-based inequalities within each ethnic group.

**Findings:**

Afrodescendants represented from 2·8% (Honduras) to 59·1% (Brazil) of the national samples. Of the 128 combinations of country and indicators with data, Afrodescendants fared worse in 78 (of which 33 were significant) and performed better in 50 (15 significant). More systematic disadvantages for Afrodescendants were found for demand for family planning satisfied, early marriage, and household handwashing and sanitation facilities. In contrast, Afrodescendants tended to present lower c-section rates and lower stunting prevalence. Honduras was the only country where Afrodescendants performed better than non-Afrodescendants in several indicators. Wealth gaps among Afrodescendants were wider than those observed for non-Afrodescendants for most indicators and across all countries.

**Interpretation:**

Gaps in health outcomes between Afrodescendants and non-Afrodescendants were observed in most countries, with more frequent disadvantages for the former although, in many cases, the gaps were reversed. Wealth inequalities within Afrodescendants tended to be wider than for non-Afrodescendants.

**Funding:**

Pan American Health Organization, Bill and Melinda Gates Foundation, and the Wellcome Trust.

**Research in context**

**Evidence before this study**

We searched PubMed for studies examining the association between ethnicity and reproductive, maternal, newborn, child, and adolescent health outcomes in multiple Latin American and Caribbean countries. Keywords included (ethnic* OR race OR afro* OR African*) AND (Latin America* OR Caribbean OR Central America OR South America) AND (women OR child*). All searches were for content published up to December 2021. We focused on publications that included at least one Afrodescendant group and provided results for multiple countries. The identified studies were focused on nutritional outcomes (such as stunting, wasting, anemia, and feeding practices) and early childhood mortality. An analysis of child nutrition in 13 Latin American and Caribbean countries showed small differences and, in a few countries, Afrodescendant children tended to be taller than the comparison group. Another study used data from Bolivia, Colombia, Guatemala, and Peru and found that after controlling for several variables, belonging to an Afrodescendant group was associated with a higher risk of child stunting and wasting, but not with a higher risk of under-five mortality and anemia. Concerning women's health, a literature review found that discrimination against ethnic minorities – including Afrodescendants – is an important driver of suboptimal health outcomes in Latin America and the Caribbean.

**Added value of this study**

Using nationally representative data from ten Latin American and Caribbean countries, we investigated inequalities among Afrodescendants and non-Afrodescendants in 17 indicators of reproductive, maternal, newborn, child, and adolescent health. Although ethnic gaps varied by country, non-Afrodescendants showed worse health outcomes than non-Afrodescendants for many indicators. We also found evidence of inequalities among different subgroups of Afrodescendants, and the presence of wider wealth-related inequalities within Afrodescendants than among non-Afrodescendants.

**Implications of all the available evidence**

This work follows up on the international call for data disaggregation (SDG 17.18), aimed at detailing health inequalities and ensuring that no one is left behind. Ethnic inequalities are currently central issues in the international human rights agenda, yet the persistent statistical invisibility of Afrodescendants reflects structural racism and represents a major obstacle towards achieving health equity goals.

Alt-text: Unlabelled box

## Introduction

The Latin America and Caribbean (LAC) region is characterized by its multi-ethnic and multicultural heritage, with Indigenous peoples and Afrodescendants comprising a significant portion of the regional population. In the Americas, the term *Afrodescendant* primarily refers to the descendants of enslaved Africans brought to the continent from the sixteenth to the late nineteenth centuries to work in plantations, mines, factories, and houses of the white and mestizo elites.^1^ Currently, there are an estimated 134 million Afrodescendants in LAC, or about 21% of the total population, with a highly uneven distribution and wide variations in size and relative share of the population across countries.^2^ In the Caribbean, the proportion of Afrodescendants is typically larger, and race dynamics tend to be different compared to Latin American countries. Despite the heterogeneity of origins and geographical distribution in the region, slavery and its legacy of racism and social exclusion are a common point for Afrodescendants across LAC countries, with ethnicity being a common reason for discrimination and undervaluing.^3^ As a consequence, inequalities based on ethnic origin, phenotypic characteristics, skin colour, and other attributes, constitute mechanisms through which discriminated groups are kept in a position of disadvantage and vulnerability, including higher rates of poverty, unemployment, and worse health outcomes compared to European descendants or even ethnically mixed groups.^4^

In 2017, the Pan American Health Organization (PAHO) Member States approved the Policy on Ethnicity and Health, embracing the need for inclusive and collaborative solutions to address ethnic gaps in health, agreeing to guarantee an intercultural approach to health and equitable treatment of Afrodescendants, Indigenous peoples, Roma populations, and members of other ethnic groups.^5^ Moreover, in some countries, progress has been made in the formulation and implementation of policies that incorporate knowledge and ancestral practices of Afrodescendant people, a respect for their autonomy, culture, and customs, and the creation of participatory scenarios conducive to equal opportunities for all.^6^, ^7^, ^8^

Ethnic inequalities are currently central issues in the international human rights agenda and the Sustainable Development Goals (SDGs).^9^ In 2013, the United Nations General Assembly proclaimed the International Decade for People of African Descent for the period 2015 to 2024 under the theme “Recognition, justice, and development”.^10^ However, the persistent invisibility of Afrodescendants in official statistics and literature on health inequalities represents a strong expression of structural racism and a huge obstacle to achieving the goals.

The COVID-19 pandemic highlighted the vulnerability of Afrodescendant groups in LAC. A report by the Economic Commission for Latin America and the Caribbean (ECLAC) using data from the Brazilian Ministry of Health showed that the Afrodescendants [Black and Brown (“*pardo*”)] in Brazil, which comprise just over half of the population, contributed 60% of COVID-19 deaths between June and July 2020.^11^ Disadvantages in antibody prevalence against SARS-CoV-2 were also documented for Afrodescendant individuals in the nationwide EPICOVID-19 study.^12^ Pooled population-based surveys carried out between May and June 2020 showed that seropositivity was 2·2 and 2·7 times higher for Black and Brown individuals, respectively, compared to Whites. The strong reductions in relative risks reported when controlling for wealth and region of the country reveal the structural disadvantages to which these groups are subjected, being poorer, living under crowded conditions, and being unable to practice social distancing.

A recent report published by the PAHO presents a comprehensive view of the situation of Afrodescendants in LAC, including perspectives of health, gender, and socioeconomic position.^13^ With very few exceptions, Afrodescendants are shown to be less likely to be formally employed, more likely-to-be poorer, and less educated compared to other population groups, except for individuals of indigenous descent. Coverage of key health interventions, however, showed less systematic patterns of disadvantage. Note that the PAHO report was based, in part, on the same set of analyses used in the present manuscript, which consists of nationally represented household surveys conducted in the region in the period 2011-2018.^13^ Other previously published multi-country studies on ethnic inequalities in health often focused on nutritional outcomes, such as stunting, wasting, anaemia, and feeding practices, and early childhood mortality, not covering important indicators and population groups.^4^^,^^14^^,^^15^

This work follows up on the international call for data disaggregation (SDG 17.18), aimed at documenting health inequalities and ensuring that no one is left behind.^16^ Considering the importance of giving statistical visibility to this group and its diversity, we investigated inequalities between Afrodescendants and a comparison group that includes non-Afrodescendants and non-Indigenous individuals in women and child health, nutrition, and wellbeing based on national surveys from LAC countries, using the available data to provide the most comprehensive perspective of ethnic inequalities in the region as possible.

## Methods

### Study design and data sources

We performed a cross-sectional multi-country analysis of ethnic inequalities using data from ten nationally representative household surveys conducted in the 2011-2019 period (median year = 2016) that included information on ethnicity for at least one group of Afrodescendant persons. The surveys included in this study were six Multiple Indicator Cluster Surveys (MICS) [from Belize (2015), Costa Rica (2018), Guyana (2019), Panama (2013), Suriname (2018), Uruguay (2012)]^17^, ^18^, ^19^, ^20^, ^21^, ^22^; two Demographic and Health Surveys (DHS) [from Colombia (2015) and Honduras (2011)],^23^^,^^24^ the National Health Survey (*Pesquisa Nacional de Saúde* – PNS) from Brazil (2019)^25^ and the National Health and Nutrition Survey (*Encuesta Nacional de Salud y Nutrición* – ENSANUT) from Ecuador (2018).^26^ All those were designed to provide estimates for a large number of health indicators using similar multistage sampling designs to select households and individuals and provide internationally comparable data.^27^^,^^28^

Analyses included children under the age of five and women of reproductive age (15-49 years). The 2019 Brazilian survey had a particular purpose, mainly focused on risk factors for chronic diseases among individuals aged 18 years or older; for this survey, only indicators related to antenatal and delivery care were available.

### Information on ethnicity

Self-reported ethnicity of women (DHS and ENSANUT) or head of household (MICS) was obtained from multiple-choice questions about ethnic group affiliation or ancestry. In Brazil, self-reported skin colour was used.

From the original response categories, women were classified as Afrodescendant or not, based upon the country-specific context, previous literature, and consultation with experts from the selected countries. The latter group, our comparison category, includes individuals of European descent, those of mixed ancestry (excluding Afrodescendant groups), or who reported not having a specific ethnic affiliation, and, in Brazil, women who reported being white, which are, in general, the wealthier segment of Latin American populations.^1^ Indigenous peoples (ranging from less than 1% in Brazil to 11% in Panama) and small minorities identified in the surveys that were not part of the comparison group were excluded from the analyses, namely, Asians in Brazil (0.9%) and Uruguay (0.5%), the *Roma* in Colombia (less than 0.1 %), and *Montubios* in Ecuador (3.7%). The classification of Afrodescendants and non-Afrodescendants (also excluding the Indigenous population) follows the criteria adopted in previous publications exploring ethnic inequalities in the region.^1^^,^^29^ Country-specific questions and categories of the original variables are presented in *Supplementary Table* S1.

For surveys in which the collected information on ethnicity related to the head of the household only, the same category was assigned to all women and children in the household. In the remaining surveys, children were classified in the same group as their mothers. Whenever sample sizes allowed, results were also reported for subgroups of the Afrodescendant category.

The percentage of Afrodescendant women ranged from 2·8% in Honduras to 59.1% in Brazil. For each country, Table 1 presents information on the year and type of survey, to whom the information on ethnicity refers, the percentage of Afrodescendant and non-Afrodescendant women, and their respective unweighted sample sizes.

**Table 1:** Percentage and sample size of Afrodescendant and non-Afrodescendant women in 10 Latin American and Caribbean countries.

Country	Year	Source	Information on ethnicity	Afrodescendants			non-Afrodescendants		
				Categories from the original variable	%	N	Categories from the original variable	%	N
Belize	2015	MICS	Head of household	*Creole; Garífuna*	37.1%	1,544	Mestizo/ Spanish/ Latin	62.9%	2,412
Brazil	2019	PNS	Woman (skin color)	*Preta; Parda*	59.1%	18,474	*Branca*	40.9%	9,270
Colombia	2015	DHS	Woman	*Afrocolombiano; Raizal from Archipelago/Palenquero from San Basilio*	9.3%	4,398	none of the above	90.7%	30,042
Costa Rica	2018	MICS	Head of household	*Negro/Afrodescendiente; Mulato(a)*	17.3%	1,402	*mestizo(a); blanco(a)*	82.7%	5,158
Ecuador	2018	ENSANUT	Woman	*Afroecuatoriano;*	4.6%	1987	*mestizo(a); blanco(a)*	95.4%	38,097
Guyana	2019	MICS	Head of household	*African/Black*	32.7%	1,717	*Mixed race*	67.3%	1,324
Honduras	2011	DHS	Woman	*Garífuna; Negro inglés*	2.8%	674	DK/none	97.2%	17,775
Panama	2013	MICS	Head of household	*Negro o afrodescendiente*	19.5%	1,542	*Otro grupo*	80.5%	5,076
Suriname	2018	MICS	Head of household	*Maroon; Creole*	42.9%	3,456	Hindustani; Javanese; Mixed ethnicity	57.1%	4,356
Uruguay	2012	MICS	Head of household	*Afro o negra*	7.5%	301	*Blanca; Otro*	92.5%	2,286

DHS: Demographic and Health Survey; ENSANUT: *Encuesta Nacional de Salud y Nutrición* (National Health and Nutrition Survey); MICS: Multiple Indicator Cluster Survey; PNS: *Pesquisa Nacional de Saúde* (National Health Survey).

### Other variables

For descriptive purposes, we estimated the percentage of women in each ethnic group by area of residence and wealth tertiles. Area of residence was defined as either urban or rural, based on the classification adopted by the country at the time of the survey. Wealth tertiles were derived from asset indices based on asset ownership (e.g. TV set), access to services (e.g. electricity), and dwelling characteristics (e.g. floor materials).^30^ A factor score generated through principal component analysis and adjusted for urban and rural residences was assigned to each household. We used the scores provided in DHS and MICS datasets and followed the same methodology for ENSANUT 2012 and PNS 2013. Household wealth scores were divided into tertiles and assigned to resident women and children; the lowest tertile included the poorest 33% of the sample, and so on. We opted to use tertiles instead of the usual quintiles due to sample size limitations when analyzing health outcomes by ethnicity.

### Outcomes

We selected 17 outcomes covering the continuum of reproductive, maternal, newborn, child, and adolescent health (RMNCAH). The selection was based on data availability and relevance, covering reproductive health, antenatal care, delivery assistance, breastfeeding and nutritional status, immunization, child protection, water, sanitation and hygiene, adolescent fertility, and early childhood mortality. We report on indicators with information for at least 50 Afrodescendants in the sample and available for at least four countries. These indicators are listed in Table 2, with detailed definitions in *Supplementary Table* S2.

**Table 2:** Definition of indicators used in the analyses.

Group	Indicator	Numerator	Denominator
Reproductive Health	Demand for family planning satisfied with modern methods	Women who are using (or whose partner is using) any modern method	Women aged 15-49 years sexually active (had sexual intercourse in the past month OR is currently married or in a union) in need of contraception
Antenatal care	Early start of antenatal care	First antenatal care visit during the first trimester of pregnancy	Women aged 15-49 years who had a birth in the two or three years preceding the survey^a^
	Antenatal care (4+ visits)	Women who had four or more antenatal visits with any provider	Women aged 15-49 years, last birth in the two or three years preceding the survey^a^
Delivery assistance	C-section delivery	Delivered by cesarean section	Last live birth in the two or three years preceding the survey^a^
	Institutional delivery	Delivered in a health facility	All live births in the two or three years preceding the survey^a^
Breastfeeding and child nutrition	Exclusive breastfeeding (0-5 months)	Breastfed exclusively (only breastmilk)	Last born aged 0-5 months, living with respondents
	Continued breastfeeding (12-15 months)	Currently breastfed	Last born aged 12-15 months, living with respondents
	Stunting (0-59 months)	Height-for-age < -2SD	Live children under five years, height measured, not flagged
Immunization	Full immunization coverage	Received 3 doses of DPT & 3 doses of polio & received measles & received BCG	All live children, 12-23/18-29/15-26 months (according to survey reference)
Child protection	Early marriage	Women who got married before 18 years of age	Women aged 20-24 years
	Birth registration	Birth registered	All live births in the five years preceding the survey from women aged 15-49 years
Water and sanitation	Improved drinking water access	Access to an improved source of drinking water in the household	All household members
	Improved sanitation	Improved sanitation (non-shared) facility	All household members
	Handwashing facility	A specific place with water and soap or other cleansing agents for handwashing	All household members
Adolescent fertility	Adolescent fertility rate	Number of births in the 1-60 months before the survey to women aged 15-19 years at the time of the birth	Number of women-years of exposure in the 1–60 months before the survey of women aged 15-19 years
Early childhood mortality	Infant mortality rate	Deaths at ages 0 to 11 months, including deaths reported at ages 0 to 99 days	Number of surviving children at beginning of specified age range during the specified period
	Under-five mortality rate	Deaths at ages 0 to 4 years, including deaths reported at ages 0 to 59 months and 0 to 99 days	Number of surviving children at beginning of specified age range during the specified period

a Births in the two years preceding the survey for MICS and PNS and in the three years preceding the survey for DHS and ENSANUT.

### Data analysis

We described the percentage of Afrodescendant women in each sample and the distribution of ethnic groups according to residence and wealth tertiles. Due to heterogeneity among countries, we opted not to provide aggregate regional estimates.

We calculated stratified point estimates and standard errors for all outcomes by country, using sample weights and accounting for complex survey design to ensure population representativeness. We followed the criteria adopted by DHS^31^ to omit coverage and prevalence estimates when the unweighted denominator contained fewer than 25 observations; for mortality and fertility rates, the thresholds were 250 children and 125 person-years of exposure, respectively.

For assessing absolute ethnic inequalities, we reported differences between Afrodescendants and non-Afrodescendants. Positive differences denoted that the outcome estimate was greater for Afrodescendants, whereas the opposite was true for non-Afrodescendants.

To summarize wealth-related inequalities within each ethnic group, the slope index of inequality (SII) was calculated for all indicators, separately for Afrodescendants and non-Afrodescendants. The SII is a measure of absolute inequality and provides the difference in percentage points (pp) or rates per thousand between the fitted values for the top and the bottom of the wealth distribution.^32^ For coverage and prevalence, the SII was calculated through a logistic regression model which uses the natural logarithm of the odds of the prevalence as the dependent variable and the fractional rank for the wealth index as the independent variable. For mortality and fertility rates, a linear regression was used instead. A difference of zero means no inequality between subgroups; greater values indicate higher levels of inequality.

All the analyses were carried out in Stata (Stata Statistical Software: Release 16. College Station, TX: StataCorp LLC).

### Ethics

All analyses relied on publicly available databases that guarantee participant anonymity. The institutions and national agencies in each country obtained ethics approval for the surveys.

### Role of the funding source

The funders of the study did not have any role in the study design, data collection, data analysis, data interpretation, or writing of the manuscript.

## Results

**Figure 1:**
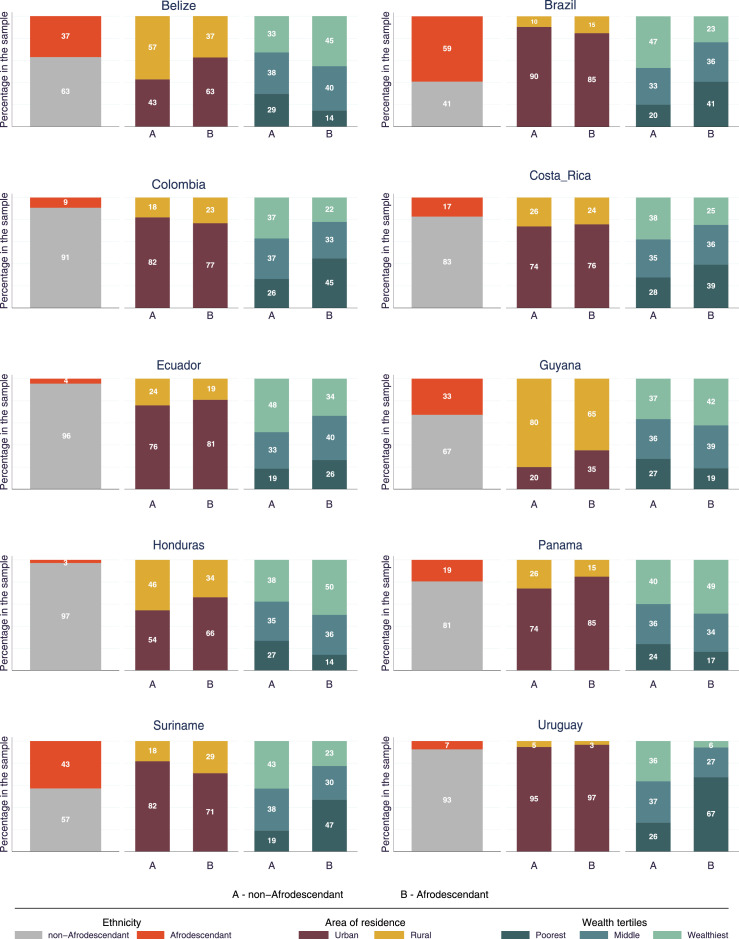
Distribution of Afrodescendants and non-Afrodescendants in ten Latin American and Caribbean countries at the national level and by area of residence and wealth tertiles.

As shown in Figure 1, Afrodescendant women were more likely to belong to the poorest wealth tertile in six of the ten countries; the highest percentage of women of African descent in the poorest group was found in Uruguay (67%) followed by Suriname (47%), Colombia (45%), and Brazil (45%). In Panama, Honduras, and Belize, more women of African descent were present in the top wealth group. Also, in most countries, Afrodescendants lived predominantly in urban areas, and in these countries, the percentage ranged from 63% in Belize to 97% in Uruguay. The only country in which a higher percentage of Afrodescendants lived in rural areas than in urban areas was Guyana, with 65%. Only in Brazil, Colombia, and Suriname, the percentage of Afrodescendants in rural areas was greater than that of non-Afrodescendants.

**Figure 2:**
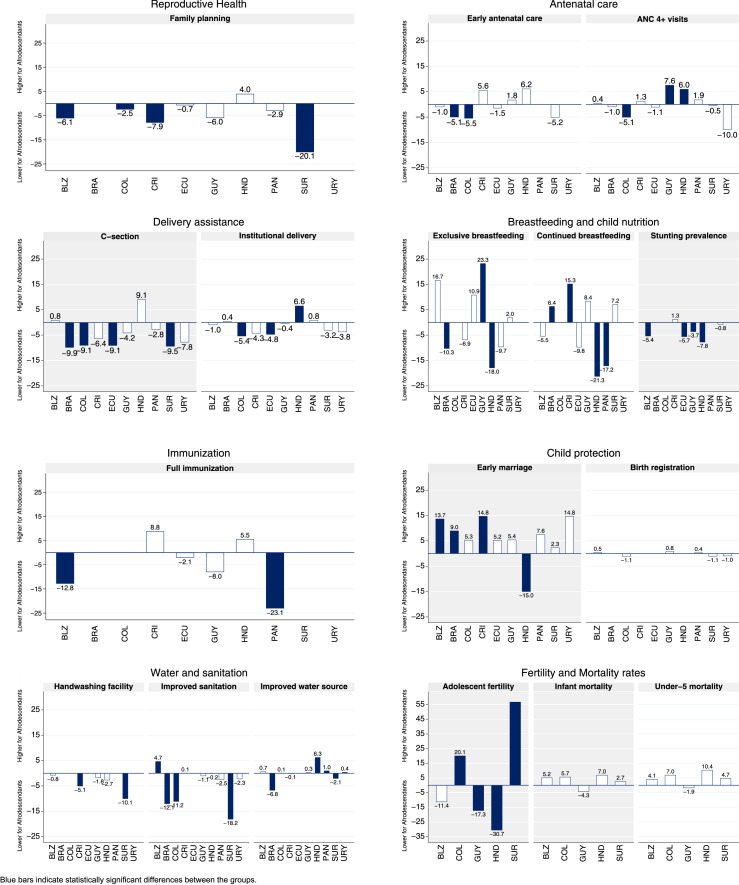
Absolute gaps between Afrodescendants and non-Afrodescendants in reproductive, maternal, neonatal, child, and adolescent health indicators, by country and indicator. Blue bars indicate statistically significant differences between the groups.

We present the estimates stratified by ethnicity for each indicator and country and the corresponding absolute inequality estimates described in the next four sections with 95% confidence intervals (95%CI) in Table 3. A visual description of absolute inequality, represented by the differences between Afrodescendants and non-Afrodescendants is presented in Figure 2. Estimates for Afrodescendants and non-Afrodescendants are presented along with 95%CI in *Supplementary Table* S2.

**Table 3:** Estimates of reproductive, maternal, neonatal, child, and adolescent health indicators for Afrodescendants and non-Afrodescendants and corresponding absolute gaps in ten Latin American and Caribbean countries.

Indicator	Country	Afrodescendants		Non-Afrodescendants		Absolute gap		
		Estimate	N	Estimate	N	Difference	95% CI	
Family planning	Belize^a^	60.8	749	67.0	1,232	-6.1	-11.6	-0.7
	Colombia^a^	83.9	1,845	86.3	18,516	-2.5	-4.8	-0.1
	Costa Rica^a^	74.0	108,672	82.0	513,148	-7.9	-13.8	-2.0
	Ecuador	76.8	1,175	77.6	23,186	-0.7	-4.7	3.3
	Guyana	38.3	816	44.3	1,683	-6.0	-12.0	0.1
	Honduras	80.0	277	76.0	9,842	4.0	-2.5	10.5
	Panama	73.9	98,435	76.8	397,488	-2.9	-9.3	3.5
	Suriname^a^	43.6	1,314	63.7	1,950	-20.1	-24.7	-15.5
Early antenatal care	Belize	69.4	200	70.4	389	-1.0	-9.8	7.8
	Brazil^a^	86.7	1,365,691	91.8	754,281	-5.1	-8.5	-1.7
	Colombia^a^	75.3	615	80.8	5,055	-5.5	-9.9	-1.1
	Costa Rica	89.9	20,569	84.3	99,049	5.6	-1.5	12.8
	Ecuador	96.1	509	97.5	9,253	-1.5	-7.2	4.2
	Guyana	51.9	233	50.1	490	1.8	-12.2	15.8
	Honduras	82.4	139	76.3	4,886	6.2	-1.4	13.8
	Suriname	54.5	554	59.6	395	-5.2	-12.7	2.4
ANC 4+ visits	Belize	94.5	200	94.1	389	0.4	-3.8	4.6
	Brazil	92.5	1,365,691	93.4	754,281	-1.0	-4.1	2.1
	Colombia^a^	86.4	615	91.6	5,055	-5.1	-8.7	-1.5
	Costa Rica	95.1	20,569	93.8	99,049	1.3	-3.1	5.7
	Ecuador	89.5	509	90.4	9,253	-1.1	-7.2	5.0
	Guyana^a^	90.3	233	82.7	490	7.6	0.7	14.5
	Honduras^a^	94.2	139	88.2	4,886	6.0	2.4	9.7
	Panama	94.5	25,496	92.6	96,643	1.9	-2.8	6.6
	Suriname	67.4	554	67.9	395	-0.5	-8.0	7.0
	Uruguay	78.0	24	88.1	192	-10.0	-30.9	10.8
C-section	Belize	36.2	200	35.4	389	0.8	-8.8	10.4
	Brazil^a^	52.2	1,365,691	62.2	754,281	-9.9	-16.4	-3.5
	Colombia^a^	38.8	665	47.9	5,369	-9.1	-15.1	-3.1
	Costa Rica	24.8	20,569	31.2	99,049	-6.4	-18.2	5.3
	Ecuador^a^	36.2	537	45.1	9,825	-9.1	-16.1	-2.2
	Guyana	20.3	233	24.5	490	-4.2	-13.6	5.2
	Honduras	28.3	154	19.2	5,294	9.1	-0.5	18.6
	Panama	30.3	25,496	33.1	96,643	-2.8	-12.8	7.1
	Suriname^a^	13.1	554	22.6	395	-9.5	-15.1	-3.9
	Uruguay	30.7	24	38.5	192	-7.8	-44.2	28.6
Institutional delivery	Belize	96.2	200	97.2	389	-1.0	-4.9	2.9
	Brazil	97.7	1,365,691	97.2	754,281	0.4	-2.3	3.2
	Colombia^a^	93.1	882	98.5	7,806	-5.4	-8.1	-2.8
	Costa Rica	94.5	20,569	98.8	99,049	-4.3	-10.4	1.8
	Ecuador^a^	93.2	881	97.7	16,651	-4.8	-8.9	-0.6
	Guyana	98.7	233	99.1	490	-0.4	-2.2	1.3
	Honduras^a^	89.7	237	83.1	8,542	6.6	1.9	11.2
	Panama	99.0	25,496	98.2	96,643	0.8	-0.5	2.1
	Suriname	92.0	554	95.2	395	-3.2	-6.8	0.4
	Uruguay	96.2	24	99.9	192	-3.8	-10.2	2.6
Exclusive breastfeeding	Belize	42.6	54	25.9	104	16.7	-5.4	38.7
	Brazil^a^	22.5	342,318	32.9	359,350	-10.3	-20.2	-0.5
	Costa Rica	16.8	3,090	23.7	25,502	-6.9	-20.0	6.2
	Ecuador	70.1	90	59.2	1,699	10.9	-5.6	27.4
	Guyana^a^	43.6	66	20.3	137	23.3	1.5	45.1
	Honduras^a^	12.1	27	30.1	849	-18.0	-29.5	-6.5
	Panama	9.1	7,706	18.8	22,650	-9.7	-24.4	5.0
	Suriname	9.6	233	7.6	124	2.0	-6.2	10.1
Continued breastfeeding	Belize	41.2	135	46.7	258	-5.5	-18.1	7.1
	Brazil^a^	44.8	1,229,335	38.4	1,023,972	6.4	0.8	12.0
	Costa Rica^a^	57.4	13,828	42.1	56,154	15.3	0.8	29.7
	Ecuador	42.3	156	52.1	3,034	-9.8	-19.6	0.1
	Guyana	54.0	153	45.6	331	8.4	-8.7	25.6
	Honduras^a^	37.4	43	58.8	1,708	-21.3	-38.1	-4.6
	Panama^a^	22.6	11,685	39.7	52,564	-17.2	-27.1	-7.3
	Suriname	23.4	397	16.3	302	7.2	-0.5	14.9
Stunting prevalence	Belize^a^	8.6	636	14.0	1,242	-5.4	-8.9	-1.9
	Costa Rica	4.7	52,575	3.4	228,182	1.3	-3.0	5.6
	Ecuador^a^	16.4	869	22.2	15,855	-5.7	-9.6	-1.7
	Guyana^a^	5.9	791	9.6	1,483	-3.7	-7.3	-0.1
	Honduras^a^	14.3	211	22.1	7,795	-7.8	-12.9	-2.7
	Suriname	7.9	1,812	8.6	1,220	-0.8	-3.9	2.4
Full immunization	Belize^a^	51.6	135	64.5	258	-12.8	-24.9	-0.8
	Costa Rica	72.7	13,828	63.9	56,154	8.8	-9.8	27.4
	Ecuador	69.2	163	71.5	3,226	-2.1	-10.2	6.1
	Guyana	58.9	153	66.9	331	-8.0	-24.2	8.2
	Honduras	90.5	45	84.9	1,801	5.5	-3.0	14.1
	Panama^a^	40.5	11,685	63.6	52,564	-23.1	-37.7	-8.5
Early marriage	Belize^a^	43.4	243	29.7	427	13.7	3.2	24.3
	Brazil^a^	25.2	4,615,270	16.2	3,066,878	9.0	4.6	13.4
	Colombia	27.6	498	22.3	5,188	5.3	-0.1	10.7
	Costa Rica^a^	29.2	32,334	14.4	158,552	14.8	5.2	24.4
	Ecuador	25.9	260	20.7	5,610	5.2	-2.0	12.5
	Guyana	35.9	322	30.4	679	5.4	-7.3	18.1
	Honduras^a^	20.7	104	35.7	3,420	-15.0	-23.9	-6.1
	Panama	28.9	26,666	21.4	108,191	7.6	-7.9	23.0
	Suriname	37.4	446	35.0	498	2.3	-5.5	10.2
	Uruguay	31.7	23	16.9	266	14.8	-13.4	43.0
Birth registration	Belize	96.2	688	95.7	1,293	0.5	-1.9	2.8
	Colombia	95.9	1,150	97.0	9,754	-1.1	-2.5	0.2
	Guyana	99.1	843	98.3	1,595	0.8	-0.6	2.2
	Panama	98.4	57,581	98.1	231,198	0.4	-1.6	2.3
	Suriname	97.8	2,284	98.9	1,648	-1.1	-2.8	0.5
	Uruguay	98.8	136	99.9	1,150	-1.0	-2.4	0.4
Handwashing facility	Belize	90.0	4,019	90.9	7,434	-0.8	-3.8	2.2
	Costa Rica^a^	84.2	623,917	89.3	3,117,329	-5.1	-8.8	-1.4
	Guyana	85.6	6,284	87.4	13,939	-1.8	-6.2	2.7
	Honduras	85.3	1,086	88.1	42,422	-2.7	-7.8	2.3
	Suriname^a^	75.1	10,374	85.1	12,220	-10.1	-13.2	-6.9
Improved sanitation	Belize^a^	92.1	4,948	87.4	8,757	4.7	1.8	7.7
	Brazil^a^	74.9	115,968,024	87.0	90,609,640	-12.1	-13.1	-11.1
	Colombia^a^	79.0	14,207	90.2	133,182	-11.2	-14.3	-8.1
	Costa Rica	93.3	735,329	93.2	3,691,061	0.1	-1.8	2.0
	Guyana	91.0	7,003	92.1	15,494	-1.1	-3.9	1.8
	Honduras	74.8	1,187	75.0	44,792	-0.2	-7.5	7.2
	Panama	89.0	648,353	91.6	2,675,740	-2.5	-7.0	2.0
	Suriname^a^	77.8	12,536	96.0	15,818	-18.2	-21.4	-15.0
	Uruguay	91.7	701	94.1	8,322	-2.3	-8.9	4.3
Improved water source	Belize	97.7	4,948	96.9	8,757	0.7	-0.8	2.3
	Brazil^a^	81.4	115,968,024	88.1	90,609,640	-6.8	-7.7	-5.9
	Colombia	93.4	14,207	93.3	133,182	0.1	-2.0	2.2
	Costa Rica	99.7	735,329	99.8	3,691,061	-0.1	-0.4	0.2
	Guyana	99.3	7,003	98.9	15,494	0.3	-0.4	1.0
	Honduras^a^	95.7	1,187	89.4	44,792	6.3	2.6	10.0
	Panama^a^	99.2	648,353	98.2	2,675,740	1.0	0.2	1.8
	Suriname^a^	97.1	12,536	99.2	15,818	-2.1	-3.2	-1.0
	Uruguay^a^	100.0	701	99.5	8,322	0.4	0.1	0.8
Adolescent fertility	Belize	75	1,326	86	2,227	-11	-31	9
	Colombia	93	2,500	73	26,193	20	8	32
	Guyana	52	1,605	70	3,109	-17	-34	-1
	Honduras	72	589	103	19,004	-31	-58	-4
	Suriname	94	2,792	37	2,969	57	44	70
Infant mortality	Belize	17	1,023	12	1,952	5	-5	15
	Colombia	20	2,263	14	18,628	6	-2	13
	Guyana	19	1,141	23	2,398	-4	-19	10
	Honduras	30	470	23	16,447	7	-10	24
	Suriname	20	2,743	17	2,197	3	-8	13
Under-5 mortality	Belize	20	1,023	16	1,952	4	-8	16
	Colombia	23	2,263	16	18,628	7	-1	15
	Guyana	22	1,141	24	2,398	-2	-17	13
	Honduras	39	470	29	16,447	10	-9	29
	Suriname	23	2,743	18	2,197	5	-7	16

Note: Estimates expressed as percentages except for adolescent fertility (births per 100,000 women aged 15-19 years) and mortality rates (deaths per 1000 live births); refer to Table 2 for a full description of the indicators.

a Indicates statistically significant differences (*p* < 0.05).

### Health and wellbeing of women

Information on demand for family planning satisfied with modern methods was available for eight countries. Afrodescendant women presented significantly lower coverage than non-Afrodescendants in four countries (Belize, Colombia, Costa Rica, and Suriname) and the largest difference was -20·1 pp in Suriname.

`Early initiation of antenatal care was less common among Afrodescendant women in five of the eight countries with data, with significant differences in Colombia and Brazil (differences of -5·5 pp and -5·1 pp, respectively).

Ten countries had information on the number of antenatal care visits. In five countries Afrodescendant women were less likely to have had at least four antenatal care visits, but only in Colombia (-5·1 pp), the difference was significant. In Guyana and Honduras, on the other hand, coverage was significantly higher among Afrodescendants (7·6 pp and 6·0 pp, respectively).

Delivery via cesarean section was less frequent among Afrodescendant women in eight out of the ten countries, with significant differences in Brazil (-9·9 pp), Suriname (-9·5 pp), Colombia (-9·1 pp), and Ecuador (-9·1 pp).

In seven countries, coverage of institutional delivery was lower for Afrodescendants, and significant differences were observed in Ecuador (-4·8 pp) and Colombia (-5·4 pp). In Honduras, the opposite was observed and Afrodescendants presented 6.6 pp higher coverage than non-Afrodescendants.

### Health, nutrition, and wellbeing of children

Exclusive breastfeeding for children under six months of age was higher for those with Afrodescendant mothers in half of the eight countries with data, but the difference was only significant in Guyana, with a difference of 23·3 pp.

On the other hand, exclusive breastfeeding was 10·0 pp and 18·0 pp lower among Afrodescendants in Brazil and Honduras, respectively. In half of the eight countries with information for continued breastfeeding at one year, it was less frequent among children from the Afrodescendant group, with large and significant differences in Honduras (-21·3 pp) and Panama (-17·2 pp). In Brazil and Costa Rica, continued breastfeeding was more frequent among Afrodescendants (6·4 and 15·3 pp higher, respectively).

Children in the Afrodescendant group were less stunted than their non-Afrodescendant counterparts in four out of the five countries with anthropometric data. Significant differences ranged from -3·7 pp in Guyana to -7·5 pp in Honduras.

Fewer children in the Afrodescendant group were fully immunized in four countries with data, but only in Belize and Panama, the difference of -12·8 pp and -23·1 pp were significant.

In nine out of the ten countries with information, point estimates for the prevalence of early marriage were higher among Afrodescendants, but only significant in Belize (13·7 pp higher), Costa Rica (14·8 pp), and Brazil (9·0 pp). In Honduras, early marriage was significantly less common among Afrodescendants with a difference of 15·0 pp below non-Afrodescendants. Small differences were found in birth registration coverage and none of them was significant.

### Access to water and sanitation

In all the five countries with information on the availability of handwashing facilities, lower coverages were found for Afrodescendants. The greater and significant differences were observed in Suriname (-10·1 pp) and Costa Rica (-5·1 pp).

A similar pattern was observed for improved sanitation and coverage for Afrodescendants was lower in seven out of nine countries with data. For this indicator, the largest and most significant differences were found in Suriname (-18·2 pp), Brazil (-12.1 pp), and Colombia (-11·2 pp).

For improved drinking water sources, the highest absolute difference was observed in Honduras, where the coverage was 6·3 pp higher in the households with Afrodescendant women. In opposition, Brazil presented a significant absolute difference of -6·8pp.

### Adolescent fertility

Adolescent fertility rates were higher for non-Afrodescendant women in three out of the five countries with information (Belize, Guyana, and Honduras) and the difference was significant in Guyana and Honduras (17 and 31 more births per thousand adolescents among non-Afrodescendants in comparison to Afrodescendants, respectively). In Colombia and Suriname, on the other hand, a significantly higher number of births from Afrodescendant adolescents were observed, with 20 and 57 more births per 1,000 women, respectively.

### Childhood mortality

There were no significant differences in the infant and under-five mortality rates of children born to Afrodescendant women and non-Afrodescendant counterparts in the five countries included in this analysis.

### Inequalities in Afrodescendant subgroups

For Costa Rica, Guyana, Panama, and Uruguay it was not possible to analyze the subgroups of Afrodescendants given that disaggregated data were not available. In Belize, no clear inequality pattern was seen between the *Creole* and *Garifuna* subgroups and the differences varied depending on the indicator analyzed. In Brazil, in general, coverage was lower for Afrodescendant women, with noticeable differences between the subgroups of *Pardas* and *Pretas*, and the latter presenting lower overall estimates. In Colombia, the *Raizal from Archipelago* and *Palanquero from San Basilio* groups were more likely to present better outcomes than *Afrocolombians*. Suriname was a country where evidence suggests more evident inequalities between Afrodescendants and non-Afrodescendants, as well as between *Maroon* and *Creole* groups, with the latter presenting more similar estimates to those from the non-Afrodescendant population. In Honduras, Afrodescendant women and children were better off than their non-Afrodescendants counterparts and the Honduran *Garífunas* seemed to be in better living conditions than the *Negro inglés* group. These estimates are presented in *Supplementary Figure* S1. Due to the small number of adolescents and children in these groups, it was not possible to present fertility and mortality data stratified by subgroups of Afrodescendants.

### Socioeconomic inequalities

**Figure 3:**
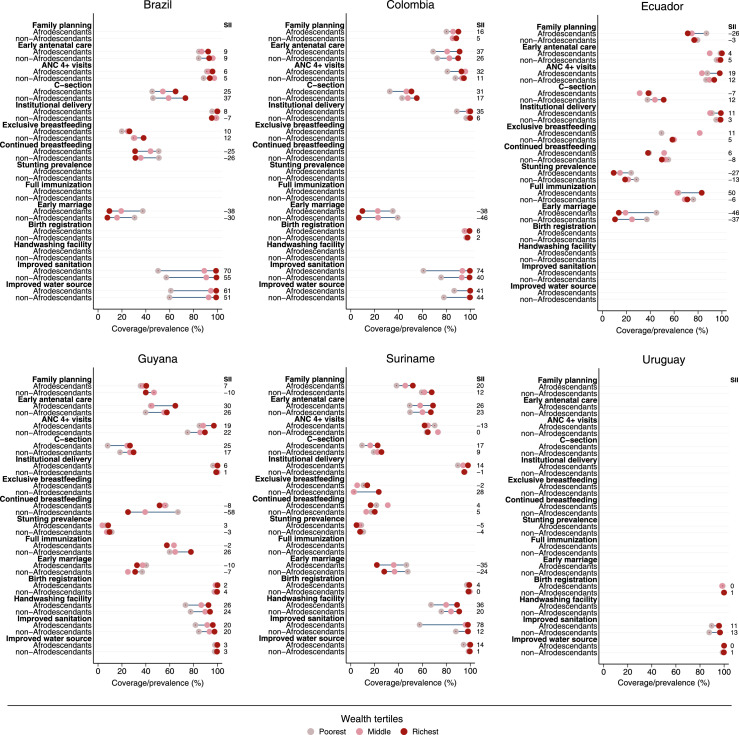
Coverage/prevalence of reproductive, maternal, neonatal, child, and adolescent health indicators by wealth tertiles for Afrodescendants and non-Afrodescendants, and the corresponding slope index of inequality (SII). South American countries.

**Figure 4:**
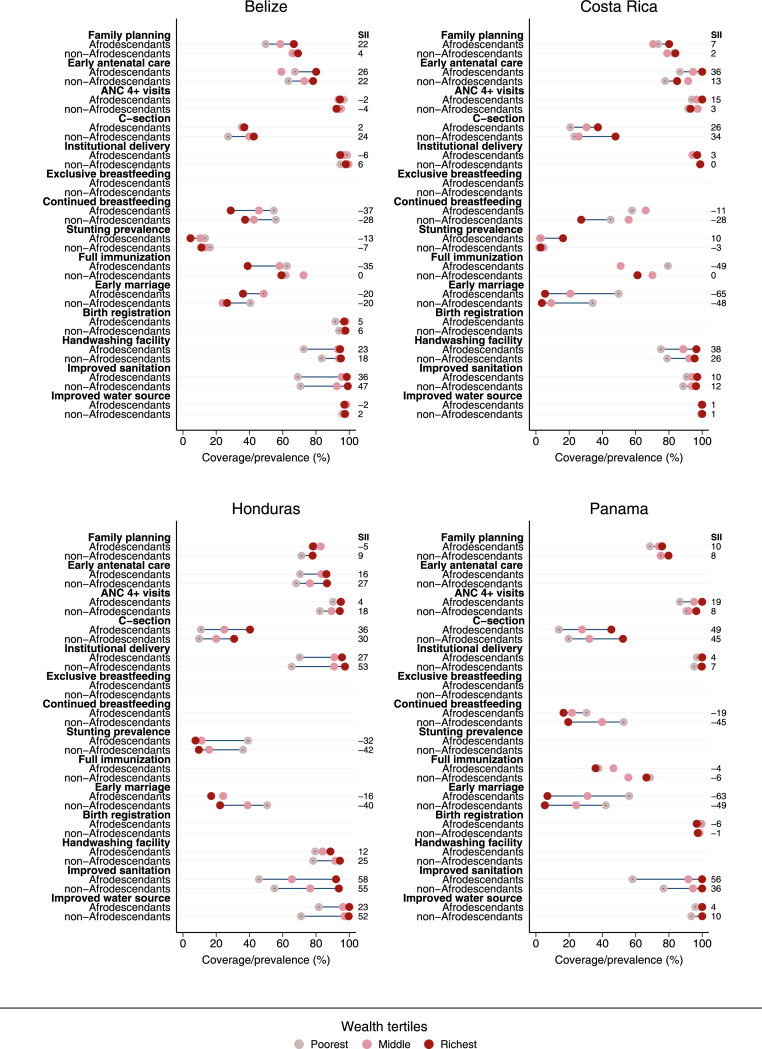
Coverage/prevalence of reproductive, maternal, neonatal, child, and adolescent health indicators by wealth tertiles for Afrodescendants and non-Afrodescendants, and the corresponding slope index of inequality (SII). Central American countries.

In Figure 3, Figure 4 we present, for South American and Central American countries separately, estimates by wealth tertiles for Afrodescendants and non-Afrodescendants, and the corresponding SII. The graphs for mortality and fertility rates are presented in *Supplementary Figure* S2. Overall, coverage of interventions was higher for wealthier individuals, for both ethnic groups, as represented by positive SII values. On the other hand, a pro-poor pattern, represented by negative SII values, was usually observed for stunting prevalence, immunization coverage, early marriage, breastfeeding indicators, early childhood mortality, and adolescent fertility rates. Nevertheless, the magnitude of the wealth-based inequality was higher among Afrodescendants for most indicators under analysis. Detailed information can be found in *Supplementary Table* S3.

## Discussion

The LAC region has been the destination of enslaved African natives during the colonial era, yet there is wide variability in the share of Afrodescendants in the countries studied (ranging from 2·8% in Honduras to 59·1% in Brazil). While it has been more than a century since slavery was abolished, Afrodescendants still lag behind both in socioeconomic and health terms. For these reasons, documenting such disparities is essential. Nevertheless, few multi-country publications addressing ethnic inequalities in women's and child's health in the region were located^13^^,^^14^^,^^33^^,^^34^ and the available literature includes studies using small samples or conducted in single municipalities or communities.^35^^,^^36^ We chose to focus on Afrodescendants given the statistical invisibility of these groups, which translated into structural racism. Despite recognizing that other groups also face discrimination, such as Indigenous peoples and other social minorities, the challenges they have to overcome, when compared to Afrodescendant groups, are well differentiated which, in turn, will translate into very different strategies when promoting public policies.

In this paper, we described indicators of the continuum of RMNCAH of Afrodescendant women and children in ten LAC countries, compared with their non-Afrodescendant counterparts. We opted for a descriptive approach to report observed gaps and found varying levels of inequality within the region, yet Afrodescendants fared worse in many indicators and countries. The main exception was Honduras where the small proportion of Afrodescendants in the sample (2·8%) presented better outcomes than the rest of the population, with significantly higher coverage of four or more antenatal care visits, institutional delivery, birth registration, improved drinking water source, and lower prevalence of early marriage, c-section, continued breastfeeding, and stunting; these results are in accordance with the finding that 50% of Afrodescendants in this country belong to the wealthiest tertile of the population, as discussed below, and are corroborated by data from other sources, such as those published by the World Bank, based on the national census, showing a lower proportion of Afrodescendants living in slums, and with higher access to sewerage, electricity, and water, as well as to telephone, television, and cell phone.^1^ This observed pattern emphasizes the importance of understanding country-specific processes of interaction of ethnic belonging, social status, and health outcomes. Health disparities were also found between different Afrodescendant subgroups in some countries. This highlights the importance of analyzing the context of each subgroup, to better understand and possibly address the factors contributing to these structural inequalities.

The indicators for which we found the more systematic disadvantages for Afrodescendants were the demand for family planning satisfied, early marriage, and household handwashing and sanitation facilities. Regarding c-section rates, all countries included in the analysis presented estimates higher than that considered acceptable by the World Health Organization (10%-15%) as there is no evidence of the benefits of caesarean delivery for women or infants who do not require the procedure.^37^ In our study, Afrodescendants presented the lower rates of c-sections, with gaps around 10 percentage points less in some countries, notably Brazil and Ecuador. This may reveal lower access to health services or discrimination since often c-section is seen as a superior alternative to vaginal birth or a medical necessity.^38^ As we observe from our findings, c-section rates were more frequent among the most privileged groups, and in our sample, in general, non-Afrodescendants are wealthier than Afrodescendants, leading to a systematic pattern of inequality. We also observed substantial disadvantages for Afrodescendants in terms of handwashing facilities and improved sanitation, particularly for Suriname and Guyana where a larger proportion of Afrodescendants than non-Afrodescendants live in rural areas.

Stunting was less common in Afrodescendants in five out of the six countries with data. Given that stunting is associated with poverty and worse living conditions,^39^ this finding is unexpected. Potential explanations include the large proportion of non-Afrodescendants with mixed European and Indigenous ancestry, as stunting is highly prevalent among Indigenous peoples.^14^ A previous study on undernutrition by ethnic groups in Latin America reinforced the scarcity of literature on this topic addressing Afrodescendants as a separate group.^14^ Studies covering ethnic inequalities in nutritional status in LAC countries often compare Indigenous and non-Indigenous peoples^40^, ^41^, ^42^ or combine Indigenous and Afrodescendants in a single group.^4^ Our findings are corroborated by studies from Colombia and Ecuador which found that Afro-Colombian and Afro-Ecuadorians presented a lower prevalence of stunting than their non-Afrodescendants counterparts as well as better growth trajectories for height-for-age and weight-for-age during the first five years of life.^43^, ^44^, ^45^ Regarding childhood mortality, we did not find significant differences and this finding is in accordance with previous studies.^4^

Our results also provided insights concerning the intersectionality between ethnicity and sociodemographic conditions. Afrodescendants were frequently poorer than their non-Afrodescendants counterparts, which could partly mediate the observed inequalities. The PAHO report on the health of Afrodescendants in the LAC region describes their socioeconomic situation, including education, employment, and wealth and shows that the economic disadvantage is more systematic than the health disadvantage.^13^ This may suggest that health systems can – at least in part – offset the socioeconomic vulnerability. Yet, this is highly country-dependent, as our results show.

In Belize, Honduras, and Panama, higher proportions of Afrodescendants belong to the wealthiest tertiles, which is reflected in higher health coverage and better housing and sanitation conditions. Afrodescendant populations in the region tend to be concentrated in urban settings, with LAC being one of the most urbanized region in the world.^1^ Among our indicators of household characteristics, improved sanitation presented the lowest coverages and the highest ethnic inequalities. Some previous evidence shows that the proportion of Afrodescendants living in slums is substantially higher than that of whites or mestizos—around twice as high in countries such as Brazil, Colombia, Costa Rica, Ecuador, Mexico, and Uruguay, which could explain some of the observed differences.^1^

An original finding in our analyses was that wealth gaps within Afrodescendants were wider than those observed for non-Afrodescendants in many cases. Therefore, not only are Afrodescendants in a worse situation in terms of coverage but also there are larger wealth inequalities among them. Wealthier Afrodescendants are usually comparable with their non-Afrodescendants counterparts while the poorest Afrodescendants lag way behind.

Some limitations in this study need to be recognized. It would have been desirable to include more countries in our analyses, but only ten out of the 33 countries from the LAC region are represented in the study (*Supplementary Figure* S3) as several surveys from countries in the region do not include questions to identify ethnic groups. Also, those with information often rely upon different questions, making comparisons difficult. It ought to be mentioned that the classification criteria for ethnic identification do not always coincide with the census criteria, nor do the classification categories, besides the limitations of the questions for ethnic identification that usually is the same for Indigenous, Afrodescendants, and other ethnic groups in the country.^46^, ^47^, ^48^ The category used for comparison (neither Afrodescendant nor Indigenous) may reflect some limitations, as mixed groups and mestizos are not homogeneous and vary in their composition. Household surveys are not designed to guarantee representativeness for different ethnic groups, which could be achieved by stratification and this issue needs to be considered in the design of future surveys. On the other side, sample sizes in some surveys were small, particularly when stratified by wealth tertiles. For MICS, the estimates were based on the ethnicity of the head of the household while for the remaining surveys the estimates were based on the women's self-identification. In both cases, children were classified according to their mother's or the head of the household's ethnicity. The proportion of non-response for ethnicity may be a limitation for inequality analysis in some countries, such as Uruguay (13% of women in the sample) and Costa Rica (7%) (*Supplementary Table* S2). The variability in the process of collecting information on ethnicity implies that the data is not perfectly comparable across countries but reinforces the demand for greater attention in this process as well as the call for standardized surveys that would allow for better comparison within the region in future analysis.

Additional limitations include the variable number of outcomes available in the included surveys and the fact that indicators such as antenatal or delivery assistance were asked for births that took place during the five years prior to the survey for DHS or two years for MICS preceding the survey, thus contributing to the time lag since these interventions took place. A related issue refers to the precision of maternal recall of events that took place several years before the interview. Also, the included surveys were carried out from 2011 until 2019, with a median year of 2016, so this must be considered when interpreting the results presented in this study.

While recent surveys are not available for all study countries, the period covered (2011-2019) provides a baseline to monitor the progress in ethnic inequalities as new surveys may become available. Given that representative, disaggregated data are essential for the design, implementation, and monitoring of actions aimed at guaranteeing the rights of people of African descent, this work contributes to the statistical visibility of Afrodescendant women and children in the region. We were also able to present separate estimates for subgroups of Afrodescendants.

In conclusion, our analyses provide a landscape view of the status and magnitude of ethnic inequalities in the LAC region for women and children, by describing multiple dimensions of health, wellbeing, and living conditions. We found that one or more health indicators were worse off among Afrodescendant people in most of the ten LAC countries analyzed. Although other social dimensions play important roles in the population's health in the region, the analyses by ethnicity allowed us to describe how Afrodescendants are affected by unequal social and health experiences when compared to a group that does not include Afrodescendants or Indigenous individuals. In this context, our results will help national policymakers target Afrodescendants with specific interventions and programs in order to achieve the SDG motto of leaving no one behind. It will also be important to monitor the impact of the COVID-19 pandemic on levels and inequalities in health status and coverage of intervention among Afrodescendant women and children, given current evidence of ethnic gaps in the impact of the pandemic.^49^ We reinforce our concern with other ethnic minorities that also suffer structural discrimination and, given their distinct realities, efforts to address inequalities in health resulting from this process should recognize the groups as distinctive collectivities. Furthermore, we reinforce the opportunity to discuss the findings from the comparison between Afrodescendants and non-Afrodescendants in more depth, which would not be possible if we have included more groups.

Our work strengthens the call for high-quality timely reliable data disaggregated by race and ethnicity and qualitative and ethnographic studies to better understand these determinants, and how they play out in subgroups. This comprises the inclusion of the ethnicity variable in health registries; the assurance that public information is accessible through culturally and adequate appropriate comunication, and that steps be taken to address the different needs of the diverse groups. These differences should be specifically addressed taking into account cultural differences and acknowledging their root in systematic oppression and racial exclusion.

## Contributors

J.C.C., A.J.D.B., G.G.D., and O.J.M. designed the study. J.C.C. and G.G.D. accessed and verified the underlying data reported in the manuscript, conducted the analysis and wrote the first draft of the manuscript. J.C.C., G.G.D., A.J.D.B, O.J.M., and C.G.V. wrote and revised the manuscript. S.P., L.C., A.S., and S.C. provided feedback and revised the text. All authors read and approved the final manuscript.

## Data sharing statement

The data are anonymized and geographically scrambled to ensure confidentiality and are publicly available through the agencies’ websites.

## Editor note

*The Lancet* Group takes a neutral position with respect to territorial claims in published maps and institutional affiliations.

## Declaration of interests

This report contains the collective views of an international group of experts and does not necessarily represent the decisions or the stated policy of the Pan American Health Organization (PAHO) or the United Nations International Children Emergency Fund (UNICEF). The authors declare no conflict of interest.

## Appendix Supplementary materials

Image, application 1
