# Supplementary material for: Inequalities in the health, nutrition, and wellbeing of Afrodescendant women and children: A cross-sectional analysis of ten Latin American and Caribbean countries
Source: Lancet Reg Health Am. 2022 Aug 19;15:100345. doi: 10.1016/j.lana.2022.100345 (PMC9669334; doi:10.1016/j.lana.2022.100345)
Supplement: Supplementary file 1 [file mmc1.docx]

Supplementary Materials

**Supplementary Table S1 – Ethnicity categories and unweighted sample sizes from the original variable available in the individual survey dataset.**

| **Survey** | **Question** | **Ethnicity** | **Unweighted sample size** | | | | **Unweighted percentage (%)** | **Non response (%)** |
| --- | --- | --- | --- | --- | --- | --- | --- | --- |
|  |  |  | **Indigenous*** | **Afrodescendant** | **non-Afrodescendant** | **Excluded** |  |  |
| Belize (2015) MICS | To what ethnic group does the head of this household belong? | Creole | 0 | 1,185 | 0 | 0 | 23.3 | 0 |
|  |  | Maya | 689 | 0 | 0 | 0 | 13.5 |  |
|  |  | Mestizo/Spanish/Latin | 0 | 0 | 2,412 | 0 | 47.3 |  |
|  |  | Garifuna | 0 | 359 | 0 | 0 | 7.1 |  |
|  |  | Other | 0 | 0 | 0 | 450 | 8.8 |  |
| Brazil (2019) PNS | Cor ou raça | Branca | 0 | 0 | 9,270 | 0 | 32.94 | 0 |
|  |  | Preta | 0 | 3,109 | 0 | 0 | 11.05 |  |
|  |  | Amarela | 0 | 0 | 0 | 197 | 0.7 |  |
|  |  | Parda | 0 | 15,365 | 0 | 0 | 54.59 |  |
|  |  | Indígena | 204 | 0 | 0 | 0 | 0.7 |  |
|  |  | Ignorado | 0 | 0 | 0 | 1 | 0 |  |
| Colombia (2015) DHS | De acuerdo con su cultura, pueblo o rasgos físicos, (NOMBRE) es o se reconoce como: | indigenous | 4,264 | 0 | 0 | 0 | 11 | 0.04 |
|  |  | gypsy (rom) | 0 | 0 | 0 | 14 | 0 |  |
|  |  | raizal from archipela | 0 | 460 | 0 | 0 | 1.2 |  |
|  |  | palanquero from san b | 0 | 72 | 0 | 0 | 0.2 |  |
|  |  | black/mulato/afro-col | 0 | 3,866 | 0 | 0 | 10 |  |
|  |  | none of the above | 0 | 0 | 30,042 | 0 | 77.6 |  |
| Costa Rica (2018) MICS | ¿(NOMBRE DEL JEFE/A DEL HOGAR) SE CONSIDERA… | Indígena | 326 | 0 | 0 | 0 | 4 | 6.8 |
|  |  | Negro(a) o ~~/~~afrodescendiente | 0 | 225 | 0 | 0 | 2.7 |  |
|  |  | Mulato(a) | 0 | 1,177 | 0 | 0 | 14.3 |  |
|  |  | Chino(a) | 0 | 0 | 0 | 20 | 0.2 |  |
|  |  | Mestizo(a) | 0 | 0 | 2,171 | 0 | 26.4 |  |
|  |  | Blanco(a) | 0 | 0 | 2,987 | 0 | 36.3 |  |
|  |  | Ninguna | 0 | 0 | 0 | 685 | 8.3 |  |
|  |  | Otro | 0 | 0 | 0 | 70 | 0.85 |  |
|  |  | No sabe, no responde | 0 | 0 | 0 | 556 | 6.8 |  |
| Ecuador (2018) ENSANUT | ¿Cómo se identifica (…) según su cultura y costumbres: | indígena | 6,818 | 0 | 0 | 0 | 14 | 0 |
|  |  | afroecuatoriano | 0 | 1987 | 0 | 0 | 4.1 |  |
|  |  | mestizo(a) | 0 | 0 | 37,454 | 0 | 76.9 |  |
|  |  | blanco(a) | 0 | 0 | 643 | 0 | 1.3 |  |
|  |  | montubio u otros | 0 | 0 | 0 | 1798 | 3.7 |  |
| Guyana (2019) MICS | TO WHICH ETHNIC GROUP DOES (name) BELONG? | East Indian | 0 | 0 | 2,262 | 0 | 34.4 | 0.3 |
|  |  | African/Black | 0 | 1,717 | 0 | 0 | 26.1 |  |
|  |  | Amerindian | 1,251 | 0 | 0 | 0 | 19 |  |
|  |  | Mixed Race | 0 | 0 | 1,324 | 0 | 20.1 |  |
|  |  | Other ethnicity/Missing/DK | 0 | 0 | 0 | 22 | 0.3 |  |
| Honduras (2011) DHS | A cuál de los siguientes grupos étnicos se considera usted que pertenece: | Garifuna | 0 | 442 | 0 | 0 | 1.9 | 0.02 |
|  |  | Negro ingles | 0 | 232 | 0 | 0 | 1.0 |  |
|  |  | Tolupan | 92 | 0 | 0 | 0 | 0.4 |  |
|  |  | Pech (paya) | 66 | 0 | 0 | 0 | 0.3 |  |
|  |  | Misquito | 773 | 0 | 0 | 0 | 3.4 |  |
|  |  | Nahoa | 8 | 0 | 0 | 0 | 0.04 |  |
|  |  | Lenca | 2,134 | 0 | 0 | 0 | 9.4 |  |
|  |  | Tawaka (sumo) | 27 | 0 | 0 | 0 | 0.1 |  |
|  |  | Maya chorti | 404 | 0 | 0 | 0 | 1.8 |  |
|  |  | Other | 0 | 0 | 0 | 799 | 3.5 |  |
|  |  | DK/none | 0 | 0 | 17,775 | 0 | 78.1 |  |
|  |  | 999 | 0 | 0 | 0 | 5 | 0.02 |  |
| Panama (2013) MICS | ¿PERTENECE A ALGÚN GRUPO INDÍGENA? | Indígena | 3,227 | 0 | 0 | 0 | 32.8 | 0 |
|  | ¿SE CONSIDERA NEGRO O AFRODESCENDIENTE? | Negro o afrodescendie | 0 | 1,542 | 0 | 0 | 15.7 |  |
|  |  | Otro grupo | 0 | 0 | 5,076 | 0 | 51.5 |  |
| Suriname (2018) MICS | To what ethnic group does the head of the household from HL2) belong? | Indigenous/Amerindian | 472 | 0 | 0 | 0 | 5.5 | 0 |
|  |  | Maroon | 0 | 2,011 | 0 | 0 | 23.6 |  |
|  |  | Creole | 0 | 1,445 | 0 | 0 | 16.9 |  |
|  |  | Hindustani | 0 | 0 | 2,191 | 0 | 25.7 |  |
|  |  | Javanese | 0 | 0 | 1,141 | 0 | 13.4 |  |
|  |  | Mixed ethnicity | 0 | 0 | 1,024 | 0 | 12 |  |
|  |  | Other | 0 | 0 | 0 | 249 | 2.9 |  |
| Uruguay (2012) MICS | ¿Cuál considera la ascendencia principal del jefe de hogar? | Afro o Negra | 0 | 301 | 0 | 0 | 9.7 | 12.6 |
|  |  | Asiática o Amarilla | 0 | 0 | 0 | 14 | 0.5 |  |
|  |  | Blanca | 0 | 0 | 2,159 | 0 | 69.5 |  |
|  |  | Indígena | 111 | 0 | 0 | 0 | 3.6 |  |
|  |  | Otro | 0 | 0 | 127 | 0 | 4.1 |  |
|  |  | No sabe | 0 | 0 | 0 | 336 | 10.8 |  |
|  |  | Missing | 0 | 0 | 0 | 55 | 1.8 |  |

*Indigenous people were not included in the analysis

DHS: Demographic and Health Survey; ENSANUT: *Encuesta Nacional de Salud y Nutrición* (National Health and Nutrition Survey); MICS: Multiple Indicator Cluster Survey; PNS: *Pesquisa Nacional de Saúde* (National Health Survey)

**Supplementary Table S2 – Estimates and absolute gap between Afrodescendants and non-Afrodescendants, by country.**

| **Country** | **Indicator** | **Afrodescendants** | | | | **Non-Afrodescendants** | | | | **Absolute gap** | | |
| --- | --- | --- | --- | --- | --- | --- | --- | --- | --- | --- | --- | --- |
|  |  | **Estimate** | **95%CI** | | **N** | **Estimate** | **95%CI** | | **N** | **Difference** | **95%CI** | |
| Belize | Family planning* | 60.8 | 56.3 | 65.2 | 749 | 67.0 | 63.5 | 70.3 | 1,232 | -6.1 | -11.6 | -0.7 |
|  | Early antenatal care | 69.4 | 61.7 | 76.1 | 200 | 70.4 | 65.1 | 75.2 | 389 | -1.0 | -9.8 | 7.8 |
|  | ANC 4+ visits | 94.5 | 90.0 | 97.1 | 200 | 94.1 | 90.9 | 96.2 | 389 | 0.4 | -3.8 | 4.6 |
|  | C-section | 36.2 | 28.8 | 44.3 | 200 | 35.4 | 30.0 | 41.1 | 389 | 0.8 | -8.8 | 10.4 |
|  | Institutional delivery | 96.2 | 91.7 | 98.3 | 200 | 97.2 | 93.8 | 98.7 | 389 | -1.0 | -4.9 | 2.9 |
|  | Exclusive breastfeeding | 42.6 | 24.4 | 63.0 | 54 | 25.9 | 17.2 | 37.0 | 104 | 16.7 | -5.4 | 38.7 |
|  | Continued breastfeeding | 41.2 | 32.2 | 50.9 | 135 | 46.7 | 38.8 | 54.8 | 258 | -5.5 | -18.1 | 7.1 |
|  | Stunting prevalence* | 8.6 | 6.3 | 11.6 | 636 | 14.0 | 12.0 | 16.3 | 1,242 | -5.4 | -8.9 | -1.9 |
|  | Full immunization* | 51.6 | 41.2 | 62.0 | 135 | 64.5 | 56.8 | 71.5 | 258 | -12.8 | -24.9 | -0.8 |
|  | Early marriage* | 43.4 | 34.7 | 52.5 | 243 | 29.7 | 24.2 | 35.9 | 427 | 13.7 | 3.2 | 24.3 |
|  | Birth registration | 96.2 | 93.5 | 97.8 | 688 | 95.7 | 94.2 | 96.9 | 1,293 | 0.5 | -1.9 | 2.8 |
|  | Handwashing facility | 90.0 | 87.5 | 92.1 | 4,019 | 90.9 | 88.8 | 92.6 | 7,434 | -0.8 | -3.8 | 2.2 |
|  | Improved sanitation* | 92.1 | 89.6 | 94.1 | 4,948 | 87.4 | 85.4 | 89.2 | 8,757 | 4.7 | 1.8 | 7.7 |
|  | Improved water source | 97.7 | 96.3 | 98.5 | 4,948 | 96.9 | 95.7 | 97.8 | 8,757 | 0.7 | -0.8 | 2.3 |
| Brazil | Early antenatal care* | 86.7 | 84.4 | 88.8 | 1,365,691 | 91.8 | 88.8 | 94.0 | 754,281 | -5.1 | -8.5 | -1.7 |
|  | ANC 4+ visits | 92.5 | 90.6 | 94.0 | 1,365,691 | 93.4 | 90.3 | 95.6 | 754,281 | -1.0 | -4.1 | 2.1 |
|  | C-section* | 52.2 | 48.6 | 55.9 | 1,365,691 | 62.2 | 56.6 | 67.4 | 754,281 | -9.9 | -16.4 | -3.5 |
|  | Institutional delivery | 97.7 | 96.6 | 98.4 | 1,365,691 | 97.2 | 92.9 | 98.9 | 754,281 | 0.4 | -2.3 | 3.2 |
|  | Exclusive breastfeeding* | 22.5 | 16.9 | 29.4 | 342,318 | 32.9 | 25.8 | 40.8 | 359,350 | -10.3 | -20.2 | -0.5 |
|  | Continued breastfeeding* | 44.8 | 41.1 | 48.5 | 1,229,335 | 38.4 | 34.4 | 42.6 | 1,023,972 | 6.4 | 0.8 | 12.0 |
|  | Early marriage* | 25.2 | 22.4 | 28.3 | 4,615,270 | 16.2 | 13.2 | 19.8 | 3,066,878 | 9.0 | 4.6 | 13.4 |
|  | Improved sanitation* | 74.9 | 73.7 | 76.1 | 115,968,024 | 87.0 | 86.1 | 87.9 | 90,609,640 | -12.1 | -13.1 | -11.1 |
|  | Improved water source* | 81.4 | 80.3 | 82.4 | 115,968,024 | 88.1 | 87.2 | 89.0 | 90,609,640 | -6.8 | -7.7 | -5.9 |
| Colombia | Family planning* | 83.9 | 81.5 | 86.0 | 1,845 | 86.3 | 85.5 | 87.1 | 18,516 | -2.5 | -4.8 | -0.1 |
|  | Early antenatal care* | 75.3 | 70.9 | 79.2 | 615 | 80.8 | 78.9 | 82.5 | 5,055 | -5.5 | -9.9 | -1.1 |
|  | ANC 4+ visits* | 86.4 | 82.8 | 89.4 | 615 | 91.6 | 90.0 | 92.9 | 5,055 | -5.1 | -8.7 | -1.5 |
|  | C-section* | 38.8 | 33.6 | 44.3 | 665 | 47.9 | 45.6 | 50.2 | 5,369 | -9.1 | -15.1 | -3.1 |
|  | Institutional delivery* | 93.1 | 90.0 | 95.3 | 882 | 98.5 | 98.2 | 98.8 | 7,806 | -5.4 | -8.1 | -2.8 |
|  | Early marriage | 27.6 | 22.8 | 32.9 | 498 | 22.3 | 20.5 | 24.1 | 5,188 | 5.3 | -0.1 | 10.7 |
|  | Birth registration | 95.9 | 94.4 | 97.0 | 1,150 | 97.0 | 96.5 | 97.4 | 9,754 | -1.1 | -2.5 | 0.2 |
|  | Improved sanitation* | 79.0 | 75.7 | 82.0 | 14,207 | 90.2 | 89.5 | 90.9 | 133,182 | -11.2 | -14.3 | -8.1 |
|  | Improved water source | 93.4 | 91.0 | 95.2 | 14,207 | 93.3 | 92.6 | 93.9 | 133,182 | 0.1 | -2.0 | 2.2 |
| Costa Rica | Family planning* | 74.0 | 68.3 | 79.1 | 108,672 | 82.0 | 79.6 | 84.2 | 513,148 | -7.9 | -13.8 | -2.0 |
|  | Early antenatal care | 89.9 | 84.5 | 93.6 | 20,569 | 84.3 | 78.1 | 89.0 | 99,049 | 5.6 | -1.5 | 12.8 |
|  | ANC 4+ visits | 95.1 | 91.0 | 97.4 | 20,569 | 93.8 | 90.1 | 96.2 | 99,049 | 1.3 | -3.1 | 5.7 |
|  | C-section | 24.8 | 16.2 | 35.9 | 20,569 | 31.2 | 25.8 | 37.2 | 99,049 | -6.4 | -18.2 | 5.3 |
|  | Institutional delivery | 94.5 | 84.2 | 98.2 | 20,569 | 98.8 | 97.5 | 99.4 | 99,049 | -4.3 | -10.4 | 1.8 |
|  | Exclusive breastfeeding | 16.8 | 8.1 | 31.7 | 3,090 | 23.7 | 15.3 | 34.9 | 25,502 | -6.9 | -20.0 | 6.2 |
|  | Continued breastfeeding* | 57.4 | 45.0 | 68.8 | 13,828 | 42.1 | 34.1 | 50.6 | 56,154 | 15.3 | 0.8 | 29.7 |
|  | Stunting prevalence | 4.7 | 1.8 | 11.6 | 52,575 | 3.4 | 2.5 | 4.6 | 228,182 | 1.3 | -3.0 | 5.6 |
|  | Full immunization | 72.7 | 55.6 | 85.0 | 13,828 | 63.9 | 53.9 | 72.7 | 56,154 | 8.8 | -9.8 | 27.4 |
|  | Early marriage* | 29.2 | 21.1 | 38.8 | 32,334 | 14.4 | 11.5 | 17.9 | 158,552 | 14.8 | 5.2 | 24.4 |
|  | Handwashing facility* | 84.2 | 80.4 | 87.3 | 623,917 | 89.3 | 87.7 | 90.7 | 3,117,329 | -5.1 | -8.8 | -1.4 |
|  | Improved sanitation | 93.3 | 91.4 | 94.8 | 735,329 | 93.2 | 92.1 | 94.1 | 3,691,061 | 0.1 | -1.8 | 2.0 |
|  | Improved water source | 99.7 | 99.3 | 99.9 | 735,329 | 99.8 | 99.6 | 99.9 | 3,691,061 | -0.1 | -0.4 | 0.2 |
| Ecuador | Family planning | 76.8 | 72.8 | 80.5 | 1,175 | 77.6 | 76.5 | 78.6 | 23,186 | -0.7 | -4.7 | 3.3 |
|  | Early antenatal care | 96.1 | 84.5 | 99.1 | 509 | 97.5 | 96.9 | 97.9 | 9,253 | -1.5 | -7.2 | 4.2 |
|  | ANC 4+ visits | 89.5 | 81.9 | 94.2 | 509 | 90.4 | 89.3 | 91.4 | 9,253 | -1.1 | -7.2 | 5.0 |
|  | C-section* | 36.2 | 29.8 | 43.2 | 537 | 45.1 | 43.2 | 47.1 | 9,825 | -9.1 | -16.1 | -2.2 |
|  | Institutional delivery* | 93.2 | 87.9 | 96.3 | 881 | 97.7 | 97.2 | 98.1 | 16,651 | -4.8 | -8.9 | -0.6 |
|  | Exclusive breastfeeding | 70.1 | 52.5 | 83.3 | 90 | 59.2 | 54.6 | 63.7 | 1,699 | 10.9 | -5.6 | 27.4 |
|  | Continued breastfeeding | 42.3 | 33.2 | 52.1 | 156 | 52.1 | 49.0 | 55.1 | 3,034 | -9.8 | -19.6 | 0.1 |
|  | Stunting prevalence* | 16.4 | 12.9 | 20.5 | 869 | 22.2 | 20.9 | 23.6 | 15,855 | -5.7 | -9.6 | -1.7 |
|  | Full immunization | 69.2 | 60.9 | 76.4 | 163 | 71.5 | 68.8 | 74.0 | 3,226 | -2.1 | -10.2 | 6.1 |
|  | Early marriage | 25.9 | 19.4 | 33.8 | 260 | 20.7 | 18.9 | 22.6 | 5,610 | 5.2 | -2.0 | 12.5 |
| Guyana | Family planning | 38.3 | 32.4 | 44.7 | 816 | 44.3 | 39.7 | 49.0 | 1,683 | -6.0 | -12.0 | 0.1 |
|  | Early antenatal care | 51.9 | 41.7 | 62.0 | 233 | 50.1 | 42.4 | 57.8 | 490 | 1.8 | -12.2 | 15.8 |
|  | ANC 4+ visits* | 90.3 | 85.7 | 93.6 | 233 | 82.7 | 76.5 | 87.6 | 490 | 7.6 | 0.7 | 14.5 |
|  | C-section | 20.3 | 13.6 | 29.2 | 233 | 24.5 | 19.2 | 30.8 | 490 | -4.2 | -13.6 | 5.2 |
|  | Institutional delivery | 98.7 | 95.1 | 99.7 | 233 | 99.1 | 98.1 | 99.6 | 490 | -0.4 | -2.2 | 1.3 |
|  | Exclusive breastfeeding* | 43.6 | 27.9 | 60.5 | 66 | 20.3 | 9.5 | 38.1 | 137 | 23.3 | 1.5 | 45.1 |
|  | Continued breastfeeding | 54.0 | 42.0 | 65.5 | 153 | 45.6 | 36.5 | 55.0 | 331 | 8.4 | -8.7 | 25.6 |
|  | Stunting prevalence* | 5.9 | 3.6 | 9.5 | 791 | 9.6 | 7.5 | 12.2 | 1,483 | -3.7 | -7.3 | -0.1 |
|  | Full immunization | 58.9 | 45.1 | 71.4 | 153 | 66.9 | 58.2 | 74.6 | 331 | -8.0 | -24.2 | 8.2 |
|  | Early marriage | 35.9 | 26.6 | 46.3 | 322 | 30.4 | 24.1 | 37.5 | 679 | 5.4 | -7.3 | 18.1 |
|  | Birth registration | 99.1 | 97.7 | 99.6 | 843 | 98.3 | 96.7 | 99.1 | 1,595 | 0.8 | -0.6 | 2.2 |
|  | Handwashing facility | 85.6 | 82.4 | 88.3 | 6,284 | 87.4 | 83.9 | 90.2 | 13,939 | -1.8 | -6.2 | 2.7 |
|  | Improved sanitation | 91.0 | 88.9 | 92.7 | 7,003 | 92.1 | 89.6 | 94.0 | 15,494 | -1.1 | -3.9 | 1.8 |
|  | Improved water source | 99.3 | 98.5 | 99.6 | 7,003 | 98.9 | 98.3 | 99.3 | 15,494 | 0.3 | -0.4 | 1.0 |
| Honduras | Family planning | 80.0 | 72.8 | 85.7 | 277 | 76.0 | 74.9 | 77.1 | 9,842 | 4.0 | -2.5 | 10.5 |
|  | Early antenatal care | 82.4 | 73.6 | 88.8 | 139 | 76.3 | 74.8 | 77.7 | 4,886 | 6.2 | -1.4 | 13.8 |
|  | ANC 4+ visits* | 94.2 | 89.6 | 96.9 | 139 | 88.2 | 87.0 | 89.3 | 4,886 | 6.0 | 2.4 | 9.7 |
|  | C-section | 28.3 | 19.9 | 38.4 | 154 | 19.2 | 17.8 | 20.7 | 5,294 | 9.1 | -0.5 | 18.6 |
|  | Institutional delivery* | 89.7 | 84.2 | 93.4 | 237 | 83.1 | 81.7 | 84.4 | 8,542 | 6.6 | 1.9 | 11.2 |
|  | Exclusive breastfeeding* | 12.1 | 4.7 | 27.9 | 27 | 30.1 | 26.9 | 33.4 | 849 | -18.0 | -29.5 | -6.5 |
|  | Continued breastfeeding* | 37.4 | 22.8 | 54.8 | 43 | 58.8 | 55.9 | 61.6 | 1,708 | -21.3 | -38.1 | -4.6 |
|  | Stunting prevalence* | 14.3 | 10.0 | 20.1 | 211 | 22.1 | 20.8 | 23.5 | 7,795 | -7.8 | -12.9 | -2.7 |
|  | Full immunization | 90.5 | 78.6 | 96.1 | 45 | 84.9 | 82.3 | 87.3 | 1,801 | 5.5 | -3.0 | 14.1 |
|  | Early marriage* | 20.7 | 13.4 | 30.4 | 104 | 35.7 | 33.4 | 38.1 | 3,420 | -15.0 | -23.9 | -6.1 |
|  | Handwashing facility | 85.3 | 79.5 | 89.7 | 1,086 | 88.1 | 87.0 | 89.1 | 42,422 | -2.7 | -7.8 | 2.3 |
|  | Improved sanitation | 74.8 | 66.8 | 81.4 | 1,187 | 75.0 | 73.4 | 76.5 | 44,792 | -0.2 | -7.5 | 7.2 |
|  | Improved water source* | 95.7 | 90.4 | 98.1 | 1,187 | 89.4 | 88.0 | 90.7 | 44,792 | 6.3 | 2.6 | 10.0 |
| Panama | Family planning | 73.9 | 67.5 | 79.4 | 98,435 | 76.8 | 73.9 | 79.5 | 397,488 | -2.9 | -9.3 | 3.5 |
|  | ANC 4+ visits | 94.5 | 88.2 | 97.5 | 25,496 | 92.6 | 89.7 | 94.8 | 96,643 | 1.9 | -2.8 | 6.6 |
|  | C-section | 30.3 | 21.3 | 41.0 | 25,496 | 33.1 | 27.7 | 39.0 | 96,643 | -2.8 | -12.8 | 7.1 |
|  | Institutional delivery | 99.0 | 97.6 | 99.6 | 25,496 | 98.2 | 96.7 | 99.0 | 96,643 | 0.8 | -0.5 | 2.1 |
|  | Exclusive breastfeeding | 9.1 | 2.4 | 28.6 | 7,706 | 18.8 | 11.3 | 29.6 | 22,650 | -9.7 | -24.4 | 5.0 |
|  | Continued breastfeeding* | 22.6 | 15.5 | 31.6 | 11,685 | 39.7 | 34.0 | 45.8 | 52,564 | -17.2 | -27.1 | -7.3 |
|  | Full immunization* | 40.5 | 27.5 | 55.0 | 11,685 | 63.6 | 57.6 | 69.2 | 52,564 | -23.1 | -37.7 | -8.5 |
|  | Early marriage | 28.9 | 16.4 | 45.8 | 26,666 | 21.4 | 17.0 | 26.5 | 108,191 | 7.6 | -7.9 | 23.0 |
|  | Birth registration | 98.4 | 95.5 | 99.5 | 57,581 | 98.1 | 96.9 | 98.8 | 231,198 | 0.4 | -1.6 | 2.3 |
|  | Improved sanitation | 89.0 | 83.6 | 92.8 | 648,353 | 91.6 | 89.6 | 93.2 | 2,675,740 | -2.5 | -7.0 | 2.0 |
|  | Improved water source* | 99.2 | 98.6 | 99.5 | 648,353 | 98.2 | 97.1 | 98.8 | 2,675,740 | 1.0 | 0.2 | 1.8 |
| Suriname | Family planning* | 43.6 | 40.1 | 47.1 | 1,314 | 63.7 | 60.7 | 66.5 | 1,950 | -20.1 | -24.7 | -15.5 |
|  | Early antenatal care | 54.5 | 49.5 | 59.4 | 554 | 59.6 | 53.6 | 65.4 | 395 | -5.2 | -12.7 | 2.4 |
|  | ANC 4+ visits | 67.4 | 62.1 | 72.3 | 554 | 67.9 | 62.2 | 73.2 | 395 | -0.5 | -8.0 | 7.0 |
|  | C-section* | 13.1 | 10.2 | 16.7 | 554 | 22.6 | 18.3 | 27.5 | 395 | -9.5 | -15.1 | -3.9 |
|  | Institutional delivery | 92.0 | 89.1 | 94.2 | 554 | 95.2 | 91.6 | 97.3 | 395 | -3.2 | -6.8 | 0.4 |
|  | Exclusive breastfeeding | 9.6 | 5.2 | 17.0 | 233 | 7.6 | 3.5 | 15.7 | 124 | 2.0 | -6.2 | 10.1 |
|  | Continued breastfeeding | 23.4 | 18.2 | 29.7 | 397 | 16.3 | 11.9 | 21.9 | 302 | 7.2 | -0.5 | 14.9 |
|  | Stunting prevalence | 7.9 | 5.7 | 10.7 | 1,812 | 8.6 | 6.8 | 10.9 | 1,220 | -0.8 | -3.9 | 2.4 |
|  | Early marriage | 37.4 | 32.2 | 42.9 | 446 | 35.0 | 29.3 | 41.2 | 498 | 2.3 | -5.5 | 10.2 |
|  | Birth registration | 97.8 | 96.0 | 98.7 | 2,284 | 98.9 | 97.3 | 99.6 | 1,648 | -1.1 | -2.8 | 0.5 |
|  | Handwashing facility* | 75.1 | 72.3 | 77.6 | 10,374 | 85.1 | 82.9 | 87.2 | 12,220 | -10.1 | -13.2 | -6.9 |
|  | Improved sanitation* | 77.8 | 74.5 | 80.8 | 12,536 | 96.0 | 95.1 | 96.7 | 15,818 | -18.2 | -21.4 | -15.0 |
|  | Improved water source* | 97.1 | 95.8 | 98.0 | 12,536 | 99.2 | 98.8 | 99.5 | 15,818 | -2.1 | -3.2 | -1.0 |
| Uruguay | ANC 4+ visits | 78.0 | 52.8 | 91.8 | 24 | 88.1 | 78.0 | 93.9 | 192 | -10.0 | -30.9 | 10.8 |
|  | C-section | 30.7 | 7.8 | 69.7 | 24 | 38.5 | 26.7 | 51.8 | 192 | -7.8 | -44.2 | 28.6 |
|  | Institutional delivery | 96.2 | 81.2 | 99.3 | 24 | 99.9 | 99.5 | 100.0 | 192 | -3.8 | -10.2 | 2.6 |
|  | Early marriage | 31.7 | 11.3 | 62.9 | 23 | 16.9 | 10.0 | 27.3 | 266 | 14.8 | -13.4 | 43.0 |
|  | Birth registration | 98.8 | 96.2 | 99.7 | 136 | 99.9 | 99.6 | 99.9 | 1,150 | -1.0 | -2.4 | 0.4 |
|  | Improved sanitation | 91.7 | 82.3 | 96.4 | 701 | 94.1 | 91.4 | 95.9 | 8,322 | -2.3 | -8.9 | 4.3 |
|  | Improved water source* | 100.0 | 99.8 | 100.0 | 701 | 99.5 | 99.0 | 99.8 | 8,322 | 0.4 | 0.1 | 0.8 |

**Supplementary Table S3 – Coverage and prevalence estimates for Afrodescendants and non-Afrodescendants by wealth tertiles and the corresponding slope index of inequality.**

| **Indicator** | **Country** | **Afrodescendants** | | | | | | | | **non-Afrodescendants** | | | | | | | |
| --- | --- | --- | --- | --- | --- | --- | --- | --- | --- | --- | --- | --- | --- | --- | --- | --- | --- |
|  |  | **Poorest** | | **Middle** | | **Wealthiest** | | **Slope index of inequality** | | **Poorest** | | **Middle** | | **Wealthiest** | | **Slope index of inequality** | |
|  |  | **Estimate** | **SE** | **Estimate** | **SE** | **Estimate** | **SE** | **Estimate** | **SE** | **Estimate** | **SE** | **Estimate** | **SE** | **Estimate** | **SE** | **Estimate** | **SE** |
| Family planning | Belize | **49.7** | 4.8 | **58.5** | 3.8 | **66.7** | 3.0 | **21.6** | 7.1 | **66.1** | 2.9 | **65.9** | 2.8 | **69.2** | 2.8 | **4.4** | 5.7 |
|  | Colombia | **80.1** | 1.7 | **85.6** | 1.8 | **90.1** | 2.5 | **15.8** | 4.9 | **85.3** | 0.6 | **85.2** | 0.6 | **88.3** | 0.8 | **4.7** | 1.5 |
|  | Costa_Rica | **73.6** | 3.8 | **70.5** | 5.1 | **80.1** | 5.6 | **6.6** | 9.4 | **83.1** | 1.5 | **78.9** | 2.0 | **83.9** | 2.3 | **2.3** | 4.1 |
|  | Ecuador | **87.0** | 2.4 | **74.8** | 3.3 | **71.4** | 3.5 | **-25.7** | 9.5 | **79.6** | 0.9 | **77.6** | 0.9 | **76.7** | 0.9 | **-3.4** | 3.0 |
|  | Guyana | **35.6** | 5.6 | **37.5** | 4.7 | **40.4** | 4.6 | **6.5** | 8.6 | **46.8** | 3.4 | **46.8** | 3.6 | **40.0** | 3.3 | **-10.2** | 5.5 |
|  | Honduras | **79.1** | 5.7 | **82.8** | 3.2 | **78.2** | 5.8 | **-4.9** | 10.8 | **71.2** | 1.0 | **78.0** | 0.9 | **77.9** | 1.0 | **9.3** | 2.0 |
|  | Panama | **68.6** | 6.3 | **73.9** | 4.8 | **75.9** | 4.0 | **9.8** | 10.5 | **75.1** | 2.6 | **75.1** | 2.1 | **79.8** | 2.5 | **7.5** | 5.3 |
|  | Suriname | **38.3** | 2.5 | **45.6** | 3.1 | **51.9** | 4.4 | **19.8** | 6.8 | **59.4** | 3.2 | **61.6** | 2.5 | **67.7** | 2.3 | **12.5** | 5.1 |
| Early antenatal care | Belize | **67.3** | 7.6 | **59.2** | 7.0 | **80.1** | 5.1 | **25.6** | 12.7 | **63.4** | 4.4 | **73.0** | 3.8 | **78.2** | 4.7 | **22.2** | 9.0 |
|  | Brazil | **84.4** | 1.5 | **86.7** | 2.2 | **92.1** | 2.2 | **9.4** | 3.6 | **84.3** | 2.5 | **96.0** | 1.5 | **93.0** | 2.3 | **9.1** | 4.8 |
|  | Colombia | **68.9** | 2.7 | **80.6** | 3.8 | **91.1** | 3.3 | **37.2** | 8.2 | **72.3** | 1.4 | **82.5** | 1.2 | **89.8** | 1.6 | **26.2** | 3.3 |
|  | Costa_Rica | **86.7** | 3.6 | **94.5** | 3.1 | **100.0** | 0.0 | **36.3** | 18.2 | **77.8** | 3.0 | **91.5** | 2.0 | **84.9** | 8.2 | **13.1** | 13.0 |
|  | Ecuador | **98.8** | 1.1 | **89.8** | 7.8 | **100.0** | 0.0 | **3.6** | 3.4 | **95.5** | 0.6 | **97.3** | 0.5 | **98.8** | 0.2 | **5.0** | 1.3 |
|  | Guyana | **44.1** | 9.1 | **44.9** | 7.0 | **64.7** | 9.1 | **30.4** | 15.3 | **39.8** | 5.3 | **55.3** | 5.7 | **57.6** | 6.4 | **26.3** | 11.3 |
|  | Honduras | **70.5** | 9.9 | **83.4** | 5.9 | **86.3** | 5.2 | **16.3** | 12.8 | **68.1** | 1.2 | **76.4** | 1.3 | **86.6** | 1.2 | **26.8** | 2.5 |
|  | Suriname | **49.3** | 3.1 | **58.0** | 4.6 | **68.8** | 6.8 | **25.6** | 9.5 | **49.6** | 5.5 | **60.0** | 4.7 | **67.0** | 5.5 | **23.4** | 10.3 |
| ANC 4+ visits | Belize | **96.7** | 2.0 | **93.5** | 2.4 | **94.4** | 3.3 | **-2.0** | 6.1 | **95.2** | 1.7 | **94.1** | 2.0 | **92.3** | 2.9 | **-4.0** | 5.1 |
|  | Brazil | **91.1** | 1.2 | **92.4** | 1.7 | **95.8** | 1.5 | **5.8** | 2.8 | **88.4** | 2.6 | **97.3** | 1.0 | **93.6** | 2.5 | **4.7** | 5.2 |
|  | Colombia | **80.8** | 2.3 | **95.8** | 1.6 | **92.6** | 3.6 | **32.3** | 9.4 | **87.5** | 1.2 | **93.1** | 0.8 | **94.7** | 1.5 | **11.4** | 2.5 |
|  | Costa_Rica | **93.7** | 2.2 | **96.7** | 2.7 | **100.0** | 0.0 | **15.0** | 12.4 | **91.5** | 2.5 | **97.3** | 0.9 | **93.0** | 3.8 | **3.3** | 6.9 |
|  | Ecuador | **87.8** | 3.1 | **83.3** | 7.9 | **98.2** | 0.8 | **18.5** | 7.5 | **86.7** | 1.1 | **89.6** | 1.0 | **93.6** | 0.6 | **11.6** | 2.6 |
|  | Guyana | **84.9** | 4.8 | **88.0** | 3.6 | **97.0** | 1.5 | **19.1** | 8.0 | **74.9** | 4.6 | **85.5** | 3.7 | **89.5** | 4.1 | **22.3** | 8.3 |
|  | Honduras | **90.2** | 4.6 | **95.1** | 2.8 | **94.9** | 2.8 | **4.2** | 6.4 | **82.3** | 1.0 | **89.3** | 1.0 | **94.3** | 0.8 | **18.2** | 2.1 |
|  | Panama | **86.6** | 6.9 | **95.1** | 3.7 | **100.0** | 0.0 | **19.1** | 9.8 | **90.5** | 2.0 | **91.9** | 2.6 | **96.5** | 1.8 | **8.1** | 3.9 |
|  | Suriname | **70.2** | 3.1 | **64.5** | 4.3 | **61.7** | 7.9 | **-12.8** | 9.1 | **63.4** | 5.1 | **72.9** | 4.3 | **64.4** | 5.3 | **0.2** | 10.4 |
| C-section | Belize | **35.4** | 8.9 | **35.8** | 6.6 | **36.9** | 6.7 | **2.2** | 15.8 | **27.1** | 3.9 | **39.9** | 5.2 | **42.6** | 4.9 | **23.8** | 9.2 |
|  | Brazil | **45.4** | 2.0 | **54.2** | 3.6 | **64.9** | 4.8 | **25.2** | 6.1 | **46.0** | 4.1 | **59.0** | 4.9 | **73.1** | 4.0 | **37.2** | 7.4 |
|  | Colombia | **32.6** | 3.3 | **46.5** | 5.1 | **50.8** | 9.9 | **30.5** | 11.8 | **42.9** | 1.5 | **47.7** | 1.7 | **55.2** | 3.0 | **17.3** | 4.6 |
|  | Costa_Rica | **20.7** | 6.0 | **30.5** | 10.3 | **37.3** | 12.7 | **25.8** | 17.4 | **23.1** | 3.4 | **25.7** | 4.2 | **47.9** | 7.3 | **33.9** | 11.3 |
|  | Ecuador | **39.3** | 5.6 | **31.2** | 5.4 | **38.5** | 7.1 | **-6.7** | 18.7 | **37.6** | 1.9 | **43.9** | 1.4 | **51.3** | 1.7 | **11.9** | 5.5 |
|  | Guyana | **8.0** | 3.1 | **24.4** | 5.3 | **26.8** | 8.6 | **25.0** | 12.5 | **18.4** | 4.1 | **26.7** | 4.8 | **29.8** | 6.3 | **16.6** | 9.7 |
|  | Honduras | **10.9** | 5.4 | **24.9** | 6.4 | **40.3** | 10.2 | **35.8** | 16.4 | **9.7** | 0.7 | **20.0** | 1.2 | **30.8** | 1.8 | **30.1** | 2.7 |
|  | Panama | **13.8** | 5.4 | **27.8** | 6.6 | **45.4** | 10.1 | **49.2** | 17.8 | **19.6** | 3.5 | **32.1** | 4.3 | **52.4** | 5.7 | **45.4** | 8.5 |
|  | Suriname | **9.4** | 2.0 | **16.2** | 3.3 | **22.4** | 6.4 | **17.2** | 6.7 | **19.2** | 3.9 | **22.3** | 3.6 | **25.6** | 4.2 | **8.8** | 7.8 |
| Institutional delivery | Belize | **98.8** | 1.2 | **96.6** | 2.4 | **94.6** | 2.8 | **-6.1** | 5.9 | **94.7** | 2.5 | **99.6** | 0.4 | **97.9** | 1.5 | **6.2** | 5.4 |
|  | Brazil | **95.5** | 1.0 | **99.4** | 0.3 | **99.9** | 0.1 | **7.7** | 2.0 | **99.2** | 0.4 | **98.6** | 0.9 | **95.2** | 2.9 | **-7.2** | 6.1 |
|  | Colombia | **88.7** | 2.3 | **98.5** | 0.8 | **100.0** | 0.0 | **34.6** | 9.5 | **96.6** | 0.4 | **99.4** | 0.2 | **99.9** | 0.1 | **5.9** | 0.9 |
|  | Costa_Rica | **94.1** | 4.5 | **94.2** | 3.7 | **97.0** | 3.0 | **3.3** | 13.2 | **98.7** | 0.5 | **98.9** | 0.7 | **98.8** | 1.1 | **0.2** | 1.7 |
|  | Ecuador | **91.7** | 2.3 | **90.1** | 4.9 | **99.1** | 0.5 | **11.5** | 5.1 | **95.2** | 0.6 | **98.2** | 0.3 | **98.9** | 0.3 | **3.1** | 1.4 |
|  | Guyana | **96.2** | 2.9 | **99.5** | 0.5 | **100.0** | 0.0 | **6.2** | 5.5 | **98.7** | 0.6 | **100.0** | 0.0 | **98.9** | 0.8 | **0.5** | 1.5 |
|  | Honduras | **70.2** | 7.1 | **91.0** | 3.1 | **95.6** | 2.8 | **26.9** | 9.5 | **65.3** | 1.3 | **90.9** | 0.8 | **97.4** | 0.4 | **52.7** | 2.2 |
|  | Panama | **96.8** | 1.6 | **99.7** | 0.3 | **100.0** | 0.0 | **4.3** | 2.3 | **95.2** | 1.6 | **100.0** | 0.0 | **99.7** | 0.3 | **7.2** | 3.0 |
|  | Suriname | **89.6** | 1.9 | **94.0** | 2.2 | **98.0** | 1.1 | **13.5** | 6.1 | **95.3** | 2.5 | **95.2** | 2.1 | **94.9** | 2.1 | **-0.6** | 4.5 |
|  | Brazil | **19.9** | 3.2 | **24.9** | 6.4 | **26.3** | 11.1 | **9.6** | 11.7 | **29.8** | 5.0 | **30.5** | 6.5 | **38.2** | 7.5 | **11.9** | 12.6 |
|  | Ecuador | **49.5** | 10.4 | **81.4** | 9.0 |  |  | **10.6** | 36.3 | **59.2** | 4.4 | **59.9** | 3.5 | **58.5** | 3.9 | **5.2** | 12.0 |
|  | Suriname | **10.6** | 4.2 | **5.3** | 3.8 | **13.8** | 8.6 | **-1.7** | 12.5 | **2.8** | 1.9 | **2.4** | 1.5 | **23.4** | 9.7 | **27.8** | 14.5 |
| Continued breastfeeding | Belize | **54.7** | 10.0 | **45.6** | 7.7 | **28.8** | 7.4 | **-37.4** | 15.2 | **55.8** | 6.4 | **42.7** | 6.7 | **37.4** | 7.0 | **-28.1** | 13.8 |
|  | Brazil | **50.8** | 2.1 | **44.0** | 4.1 | **31.0** | 4.1 | **-24.5** | 5.9 | **50.9** | 3.4 | **35.8** | 3.6 | **31.3** | 3.8 | **-26.0** | 6.8 |
|  | Costa_Rica | **57.8** | 7.4 | **66.0** | 11.6 |  |  | **-10.9** | 30.8 | **44.9** | 4.8 | **55.8** | 7.0 | **27.2** | 7.3 | **-27.6** | 13.7 |
|  | Ecuador | **39.0** | 8.0 | **51.6** | 7.6 | **38.1** | 10.4 | **6.1** | 33.4 | **55.1** | 2.5 | **52.6** | 2.5 | **49.7** | 2.7 | **-8.0** | 8.9 |
|  | Guyana | **56.6** | 7.1 | **55.6** | 10.1 | **51.5** | 11.0 | **-8.4** | 21.5 | **66.9** | 7.9 | **39.4** | 7.9 | **25.1** | 6.8 | **-57.8** | 12.4 |
|  | Panama | **30.5** | 9.0 | **21.8** | 7.6 | **16.6** | 7.1 | **-19.3** | 15.8 | **52.8** | 4.5 | **39.8** | 5.3 | **19.5** | 4.5 | **-44.6** | 8.2 |
|  | Suriname | **21.4** | 3.6 | **30.9** | 5.9 | **16.6** | 7.0 | **3.5** | 10.9 | **17.2** | 4.5 | **12.9** | 3.0 | **20.1** | 5.9 | **4.5** | 10.4 |
| Stunting prevalence | Belize | **13.2** | 2.9 | **10.4** | 2.5 | **4.5** | 1.3 | **-12.8** | 4.4 | **16.3** | 1.7 | **13.3** | 2.0 | **11.1** | 2.1 | **-7.4** | 4.0 |
|  | Costa_Rica | **3.1** | 1.6 | **2.5** | 1.6 | **16.3** | 12.6 | **10.1** | 11.5 | **4.8** | 0.9 | **2.1** | 0.5 | **2.9** | 1.2 | **-3.5** | 2.5 |
|  | Ecuador | **24.0** | 3.8 | **14.7** | 2.7 | **9.4** | 2.6 | **-27.2** | 9.3 | **28.4** | 1.2 | **21.6** | 1.0 | **18.9** | 1.1 | **-13.0** | 3.6 |
|  | Guyana | **6.3** | 2.3 | **3.6** | 1.4 | **8.5** | 2.7 | **3.5** | 4.8 | **11.5** | 2.4 | **7.3** | 2.3 | **9.6** | 2.3 | **-3.1** | 5.0 |
|  | Honduras | **39.1** | 8.8 | **11.2** | 3.1 | **7.5** | 4.0 | **-32.3** | 12.0 | **36.1** | 1.1 | **15.7** | 0.9 | **9.5** | 0.9 | **-41.6** | 2.2 |
|  | Suriname | **8.8** | 1.7 | **7.2** | 2.1 | **4.8** | 2.6 | **-5.4** | 4.9 | **10.5** | 1.7 | **8.1** | 1.8 | **7.7** | 1.9 | **-3.8** | 3.7 |
| Full immunization | Belize | **62.5** | 10.3 | **58.1** | 7.9 | **38.9** | 8.6 | **-35.4** | 17.9 | **61.8** | 7.0 | **72.5** | 6.3 | **59.2** | 6.8 | **0.1** | 15.0 |
|  | Costa_Rica | **79.4** | 7.7 | **50.9** | 14.8 |  |  | **-49.1** | 19.2 | **60.9** | 5.3 | **70.1** | 5.8 | **61.1** | 10.9 | **0.0** | 18.0 |
|  | Ecuador | **63.7** | 7.6 | **62.7** | 7.2 | **83.1** | 6.1 | **49.7** | 18.4 | **76.0** | 2.0 | **68.8** | 2.1 | **71.0** | 2.4 | **-6.5** | 7.4 |
|  | Guyana | **57.7** | 7.7 | **63.5** | 9.7 | **57.6** | 11.8 | **-1.7** | 21.7 | **59.9** | 5.0 | **64.5** | 7.1 | **77.7** | 7.2 | **25.6** | 12.1 |
|  | Panama | **37.7** | 10.4 | **46.6** | 11.7 | **35.9** | 11.1 | **-4.1** | 23.1 | **68.6** | 4.3 | **55.6** | 4.9 | **66.4** | 7.0 | **-6.4** | 11.5 |
| Early marriage | Belize | **48.5** | 11.4 | **48.6** | 6.5 | **36.1** | 5.8 | **-20.2** | 14.5 | **40.5** | 5.4 | **23.8** | 4.1 | **26.5** | 4.7 | **-19.9** | 9.7 |
|  | Brazil | **37.5** | 2.3 | **19.4** | 2.1 | **9.5** | 3.0 | **-38.3** | 4.8 | **30.6** | 3.9 | **15.9** | 2.9 | **7.8** | 2.1 | **-29.9** | 5.8 |
|  | Colombia | **35.1** | 3.7 | **22.6** | 4.2 | **9.7** | 3.7 | **-38.2** | 9.4 | **39.2** | 1.8 | **22.8** | 1.5 | **6.7** | 1.4 | **-46.4** | 3.1 |
|  | Costa_Rica | **49.7** | 7.0 | **20.7** | 6.3 | **5.5** | 3.3 | **-65.0** | 10.2 | **34.1** | 3.4 | **9.2** | 1.9 | **3.6** | 1.4 | **-47.8** | 5.8 |
|  | Ecuador | **45.2** | 7.4 | **19.3** | 4.4 | **13.7** | 5.1 | **-46.5** | 17.6 | **37.1** | 2.1 | **24.7** | 1.7 | **10.5** | 1.2 | **-37.2** | 4.6 |
|  | Guyana | **40.4** | 6.7 | **37.3** | 7.7 | **32.6** | 6.9 | **-10.3** | 12.3 | **36.9** | 4.7 | **25.0** | 3.9 | **31.1** | 6.8 | **-7.0** | 9.3 |
|  | Panama | **56.1** | 15.4 | **31.0** | 7.6 | **6.9** | 3.6 | **-63.1** | 15.8 | **42.0** | 4.5 | **24.2** | 4.0 | **5.4** | 2.1 | **-48.7** | 5.8 |
|  | Suriname | **46.5** | 4.3 | **35.9** | 5.2 | **21.8** | 4.7 | **-34.9** | 9.8 | **47.7** | 7.3 | **36.6** | 4.7 | **28.0** | 4.7 | **-24.0** | 10.7 |
| Birth registration | Belize | **91.7** | 3.1 | **97.8** | 0.7 | **96.9** | 2.0 | **5.4** | 4.7 | **93.9** | 1.4 | **96.4** | 1.1 | **97.7** | 1.2 | **5.7** | 2.9 |
|  | Colombia | **95.1** | 0.9 | **95.7** | 1.3 | **99.4** | 0.5 | **6.0** | 3.0 | **96.6** | 0.4 | **96.9** | 0.4 | **97.8** | 0.4 | **1.6** | 0.8 |
|  | Guyana | **99.0** | 1.0 | **98.3** | 0.8 | **100.0** | 0.0 | **1.6** | 1.4 | **97.3** | 0.9 | **98.1** | 1.6 | **99.7** | 0.3 | **3.5** | 1.6 |
|  | Panama | **99.6** | 0.2 | **99.3** | 0.5 | **96.7** | 2.3 | **-5.7** | 5.5 | **98.4** | 0.5 | **98.3** | 0.8 | **97.3** | 1.2 | **-1.5** | 1.9 |
|  | Suriname | **97.1** | 0.9 | **98.6** | 1.3 | **99.1** | 0.6 | **3.7** | 2.8 | **98.2** | 0.9 | **99.6** | 0.3 | **98.5** | 1.2 | **0.1** | 2.2 |
|  | Uruguay | **98.9** | 0.8 | **98.4** | 1.8 |  |  | **0.2** | 2.5 | **99.7** | 0.2 | **100.0** | 0.0 | **100.0** | 0.0 | **0.6** | 0.3 |
| Handwashing facility | Belize | **72.6** | 3.7 | **93.1** | 1.2 | **94.5** | 2.0 | **23.4** | 5.6 | **83.5** | 2.1 | **93.8** | 1.3 | **95.0** | 1.2 | **17.8** | 3.6 |
|  | Costa_Rica | **75.2** | 2.7 | **88.6** | 3.1 | **96.5** | 1.7 | **37.6** | 7.1 | **78.9** | 1.6 | **92.1** | 0.9 | **95.5** | 0.9 | **25.9** | 3.2 |
|  | Guyana | **73.2** | 3.6 | **86.4** | 2.1 | **92.4** | 1.8 | **26.5** | 5.9 | **77.5** | 3.2 | **89.7** | 1.8 | **93.8** | 1.2 | **23.5** | 5.0 |
|  | Honduras | **79.5** | 8.5 | **83.9** | 3.9 | **88.7** | 3.2 | **11.6** | 9.7 | **78.3** | 1.1 | **91.6** | 0.7 | **94.4** | 0.7 | **24.6** | 2.0 |
|  | Suriname | **67.1** | 1.8 | **79.7** | 2.5 | **88.9** | 2.1 | **35.8** | 4.7 | **75.8** | 2.4 | **83.9** | 1.8 | **90.7** | 1.2 | **20.4** | 3.5 |
| Improved sanitation | Belize | **69.0** | 4.4 | **95.5** | 1.0 | **98.5** | 0.8 | **36.1** | 6.6 | **70.7** | 1.9 | **92.5** | 1.2 | **99.1** | 0.3 | **47.5** | 3.2 |
|  | Brazil | **50.3** | 0.9 | **88.9** | 0.5 | **98.8** | 0.2 | **69.7** | 1.0 | **57.1** | 1.1 | **90.6** | 0.5 | **99.1** | 0.1 | **55.1** | 1.3 |
|  | Colombia | **60.8** | 2.6 | **93.4** | 1.0 | **99.7** | 0.2 | **74.3** | 3.6 | **75.5** | 0.8 | **92.9** | 0.5 | **99.4** | 0.1 | **40.4** | 1.5 |
|  | Costa_Rica | **90.6** | 1.4 | **93.9** | 1.6 | **97.0** | 1.1 | **10.2** | 3.5 | **88.6** | 0.9 | **93.8** | 0.8 | **96.3** | 0.7 | **11.9** | 1.9 |
|  | Guyana | **81.6** | 3.3 | **91.4** | 1.8 | **96.1** | 0.9 | **20.4** | 4.9 | **84.4** | 1.7 | **93.3** | 1.8 | **97.5** | 0.8 | **19.7** | 2.8 |
|  | Honduras | **45.7** | 8.9 | **65.5** | 6.9 | **92.0** | 3.1 | **58.1** | 9.1 | **55.0** | 1.4 | **76.6** | 1.2 | **93.8** | 0.7 | **54.8** | 2.0 |
|  | Panama | **58.1** | 6.6 | **91.7** | 2.3 | **99.9** | 0.1 | **55.5** | 7.9 | **76.8** | 2.5 | **94.4** | 1.0 | **99.9** | 0.0 | **36.3** | 3.9 |
|  | Suriname | **57.6** | 2.9 | **96.0** | 1.2 | **98.0** | 0.8 | **77.9** | 4.1 | **87.9** | 1.3 | **97.7** | 0.5 | **98.1** | 0.5 | **12.0** | 2.0 |
|  | Uruguay | **89.7** | 4.9 | **95.5** | 2.3 | **95.9** | 3.6 | **11.5** | 10.9 | **87.7** | 3.1 | **96.3** | 1.2 | **96.7** | 0.9 | **12.6** | 4.8 |
| Improved water source | Belize | **98.0** | 1.0 | **98.2** | 0.5 | **97.1** | 1.1 | **-1.7** | 2.3 | **95.7** | 1.2 | **97.7** | 0.6 | **97.3** | 0.7 | **2.4** | 2.1 |
|  | Brazil | **60.9** | 0.9 | **94.5** | 0.3 | **98.9** | 0.1 | **61.3** | 1.2 | **59.8** | 1.1 | **92.6** | 0.5 | **98.9** | 0.2 | **51.0** | 1.5 |
|  | Colombia | **86.5** | 2.1 | **99.7** | 0.2 | **100.0** | 0.0 | **41.0** | 6.6 | **77.9** | 1.0 | **99.3** | 0.2 | **99.8** | 0.1 | **44.2** | 2.2 |
|  | Costa_Rica | **99.4** | 0.3 | **99.9** | 0.1 | **100.0** | 0.0 | **1.0** | 0.6 | **99.3** | 0.2 | **100.0** | 0.0 | **100.0** | 0.0 | **1.0** | 0.3 |
|  | Guyana | **97.9** | 1.0 | **99.5** | 0.3 | **99.9** | 0.1 | **2.7** | 1.3 | **97.6** | 0.5 | **99.4** | 0.4 | **99.6** | 0.3 | **2.8** | 1.0 |
|  | Honduras | **81.7** | 7.9 | **96.2** | 2.2 | **100.0** | 0.0 | **23.0** | 10.2 | **71.0** | 1.6 | **97.0** | 0.4 | **99.7** | 0.1 | **52.2** | 2.7 |
|  | Panama | **95.9** | 1.2 | **99.8** | 0.2 | **100.0** | 0.0 | **4.5** | 1.4 | **93.4** | 1.4 | **99.9** | 0.1 | **100.0** | 0.0 | **10.1** | 2.6 |
|  | Suriname | **94.4** | 1.0 | **99.4** | 0.3 | **100.0** | 0.0 | **13.9** | 3.1 | **98.6** | 0.5 | **99.1** | 0.3 | **99.6** | 0.2 | **1.5** | 0.7 |
|  | Uruguay | **100.0** | 0.0 | **100.0** | 0.0 | **100.0** | 0.0 | **0.1** | 0.1 | **98.7** | 0.6 | **100.0** | 0.0 | **99.8** | 0.2 | **1.4** | 0.9 |
| Adolescent fertility | Belize | **137.4** | 18.0 | **72.5** | 11.0 | **48.5** | 8.8 | **-128.4** | 27.5 | **126.0** | 21.6 | **78.2** | 13.8 | **52.2** | 12.0 | **-86.5** | 26.6 |
|  | Colombia | **124.5** | 4.8 | **75.8** | 4.2 | **24.6** | 2.5 | **-144.6** | 0.2 | **134.5** | 11.2 | **66.5** | 9.1 | **18.4** | 8.8 | **-173.1** | 7.5 |
|  | Guyana | **123.3** | 14.0 | **64.2** | 9.6 | **32.4** | 7.4 | **-131.6** | 20.8 | **81.5** | 18.7 | **60.6** | 16.4 | **31.1** | 9.0 | **-72.9** | 0.4 |
|  | Honduras | **157.9** | 5.2 | **101.8** | 3.8 | **63.8** | 4.4 | **-134.8** | 16.9 |  |  | **91.4** | 22.5 | **43.0** | 12.8 | **-111.6** | 0.0 |
|  | Suriname | **82.2** | 12.5 | **38.2** | 7.6 | **16.1** | 4.2 | **-84.2** | 24.2 | **142.9** | 9.6 | **74.6** | 13.5 | **24.6** | 9.5 | **-179.5** | 1.0 |
| Infant mortality | Belize | **11.3** | 4.1 | **10.9** | 4.9 | **16.0** | 6.1 | **5.8** | 4.1 | **46.6** | 16.9 | **9.6** | 4.9 | **5.7** | 2.5 | **-53.3** | 25.4 |
|  | Colombia | **19.9** | 2.3 | **12.6** | 1.9 | **8.0** | 2.4 | **-17.7** | 1.4 | **20.4** | 3.6 | **21.0** | 6.7 | **13.9** | 8.1 | **-5.6** | 7.0 |
|  | Guyana | **26.2** | 7.5 | **30.8** | 9.4 | **10.5** | 3.7 | **-22.2** | 15.7 | **16.0** | 7.8 | **28.1** | 10.4 | **9.7** | 6.4 | **-11.1** | 20.5 |
|  | Suriname | **16.2** | 7.2 | **23.9** | 8.7 | **9.9** | 4.2 | **-12.9** | 15.1 | **18.1** | 3.8 | **20.5** | 7.3 | **24.4** | 12.5 | **7.8** | 2.7 |
| Under-5 mortality | Belize | **14.1** | 4.3 | **18.3** | 7.8 | **16.0** | 6.1 | **3.8** | 4.3 | **57.3** | 17.5 | **9.6** | 4.9 | **5.7** | 2.5 | **-67.0** | 33.5 |
|  | Colombia | **22.9** | 2.4 | **13.8** | 1.9 | **10.3** | 2.6 | **-18.8** | 3.3 | **25.5** | 4.3 | **22.6** | 6.8 | **13.9** | 8.1 | **-13.5** | 7.1 |
|  | Guyana | **29.4** | 7.9 | **30.8** | 9.4 | **10.5** | 3.7 | **-27.2** | 13.8 | **22.1** | 8.8 | **28.1** | 10.4 | **15.1** | 8.3 | **-11.3** | 12.7 |
|  | Suriname | **21.0** | 8.4 | **24.3** | 8.7 | **9.9** | 4.2 | **-18.9** | 11.9 | **22.3** | 4.5 | **21.4** | 7.3 | **28.3** | 12.8 | **4.4** | 7.0 |


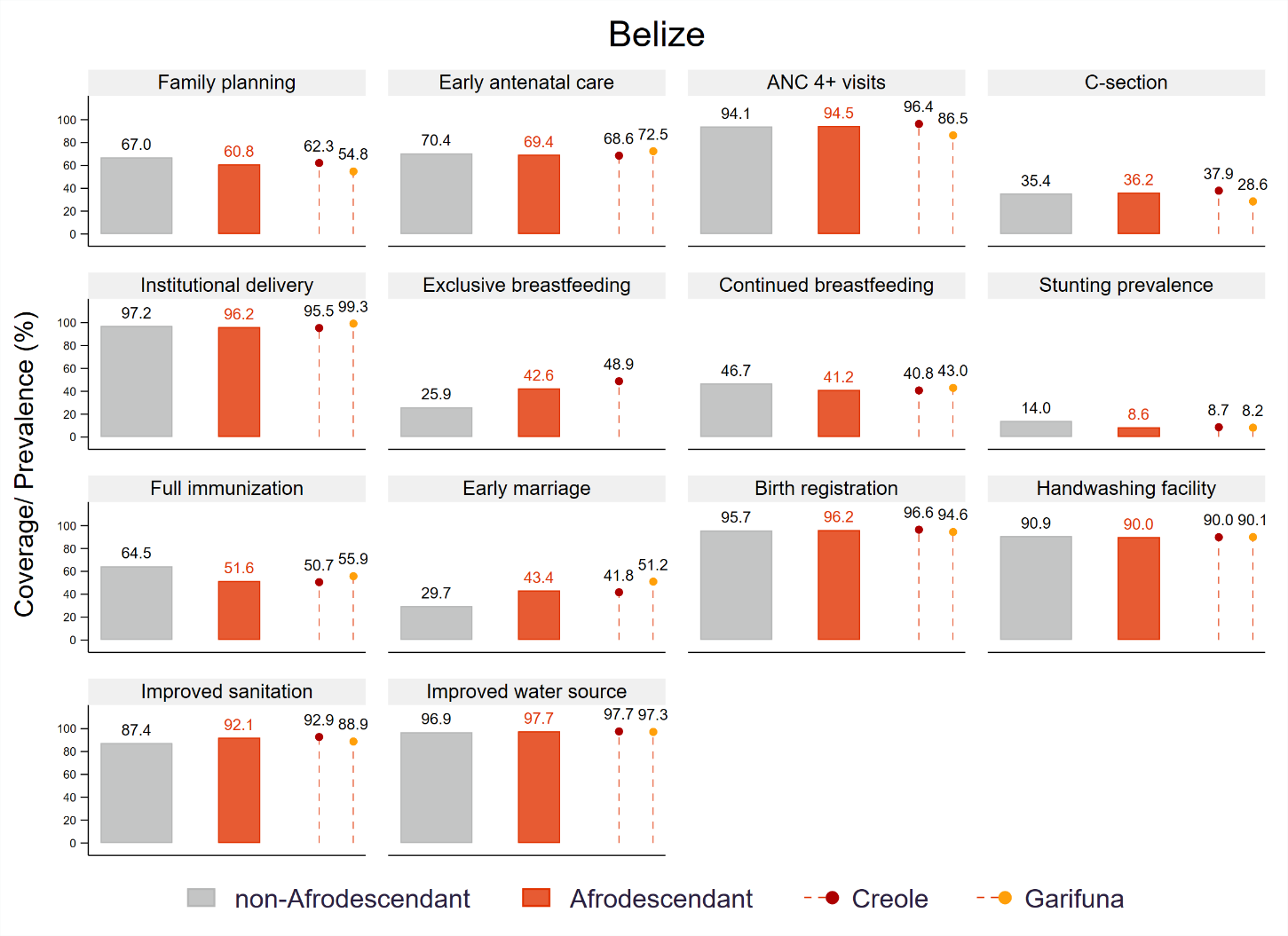

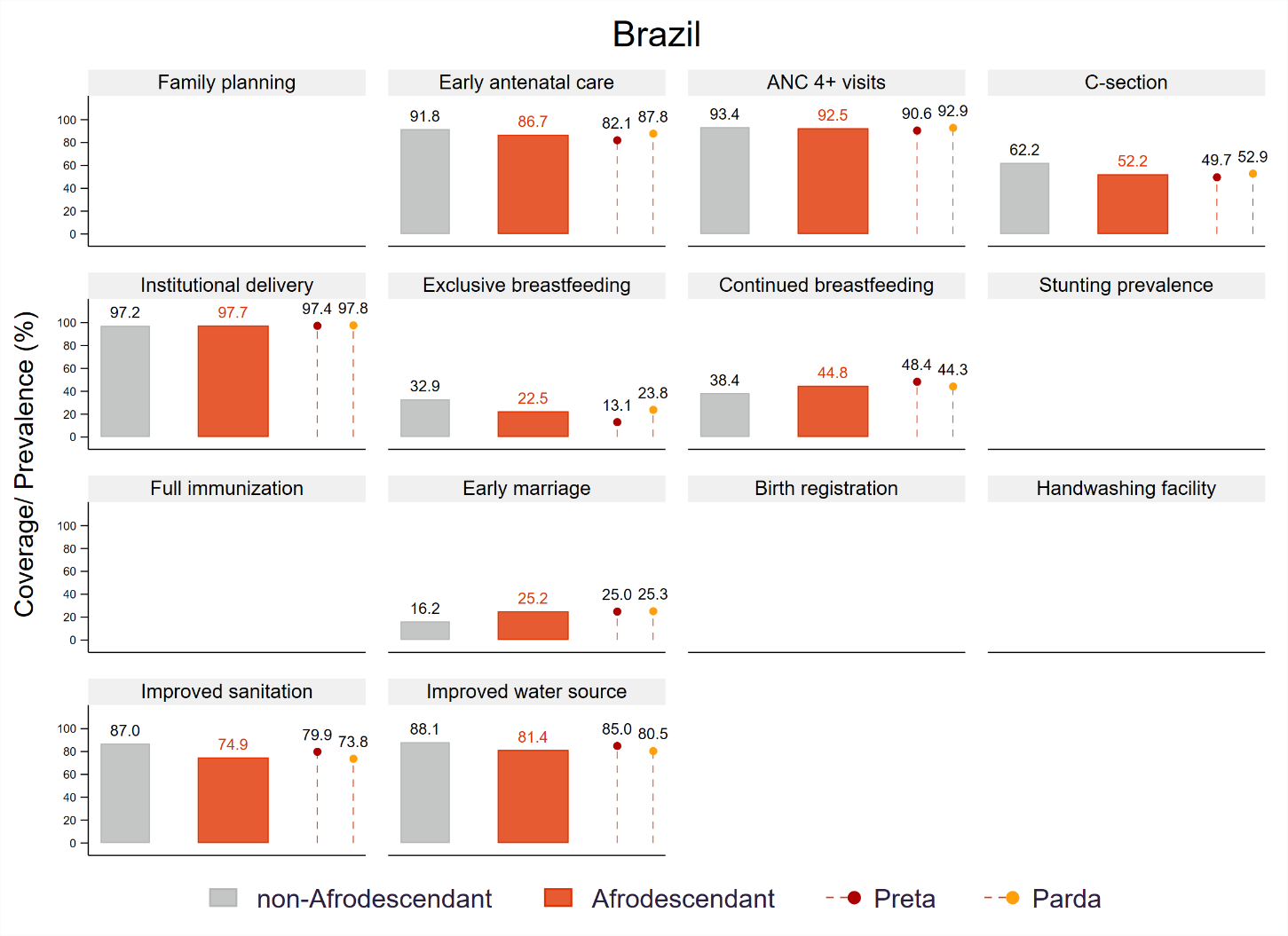

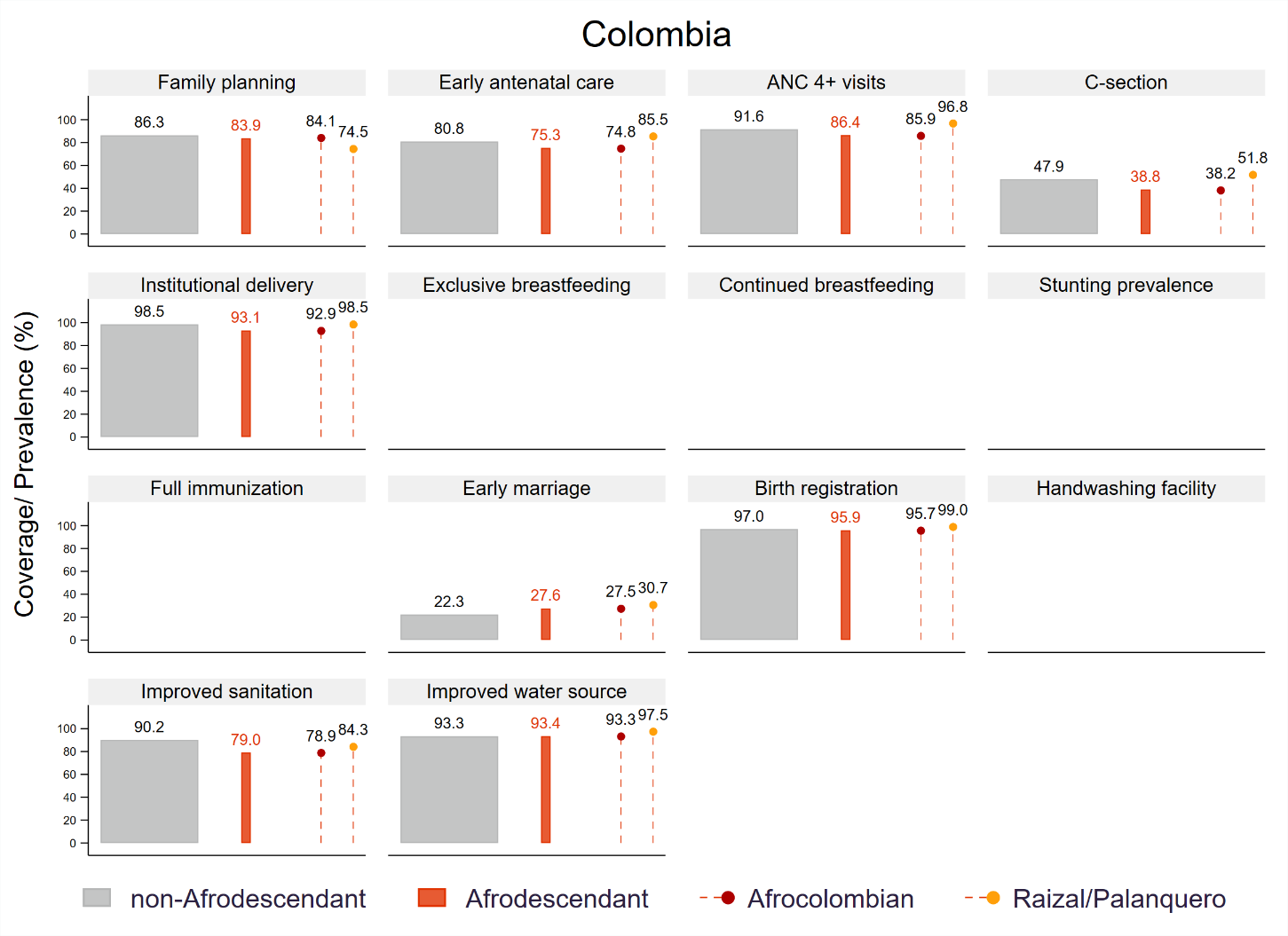

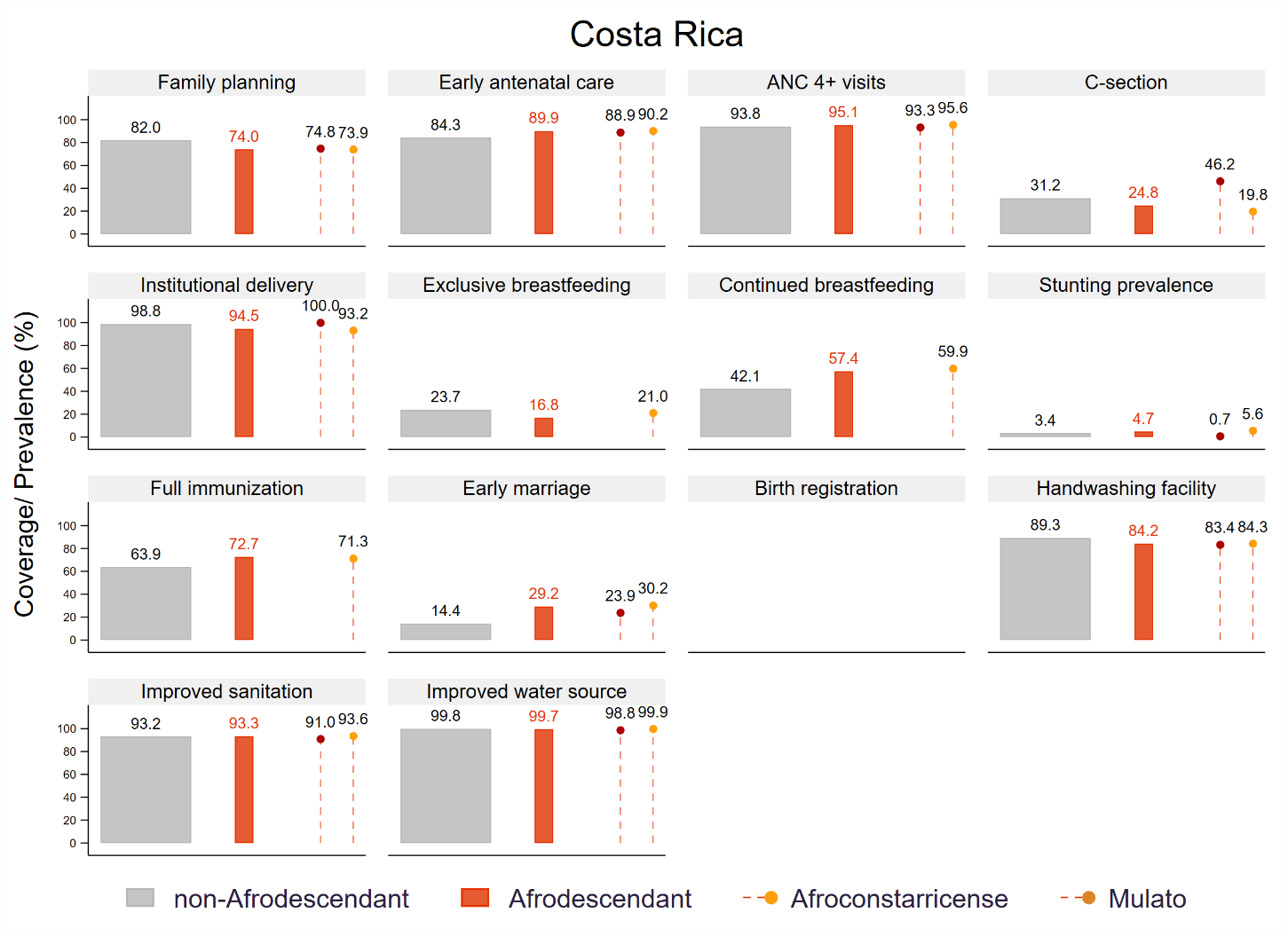

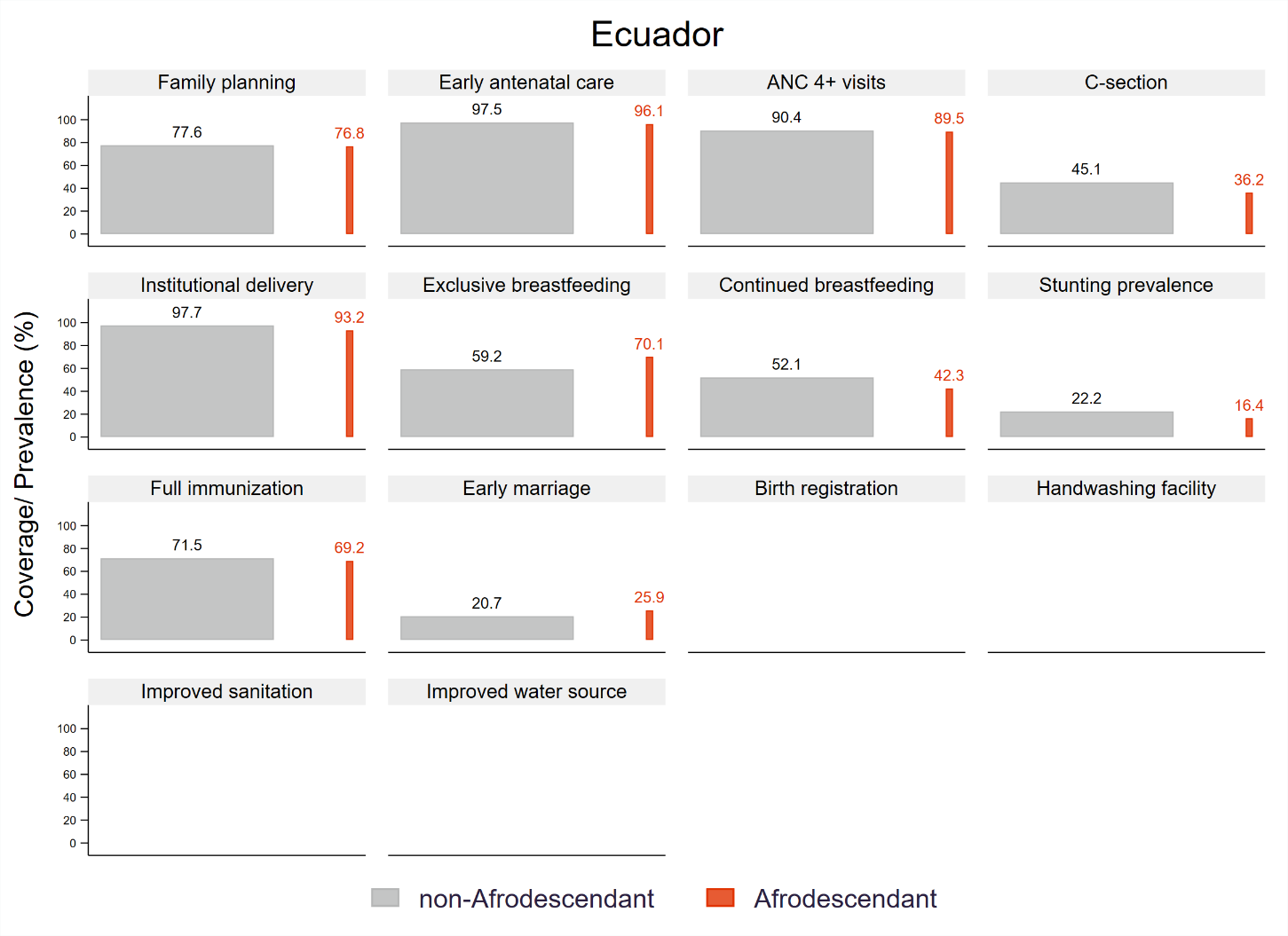

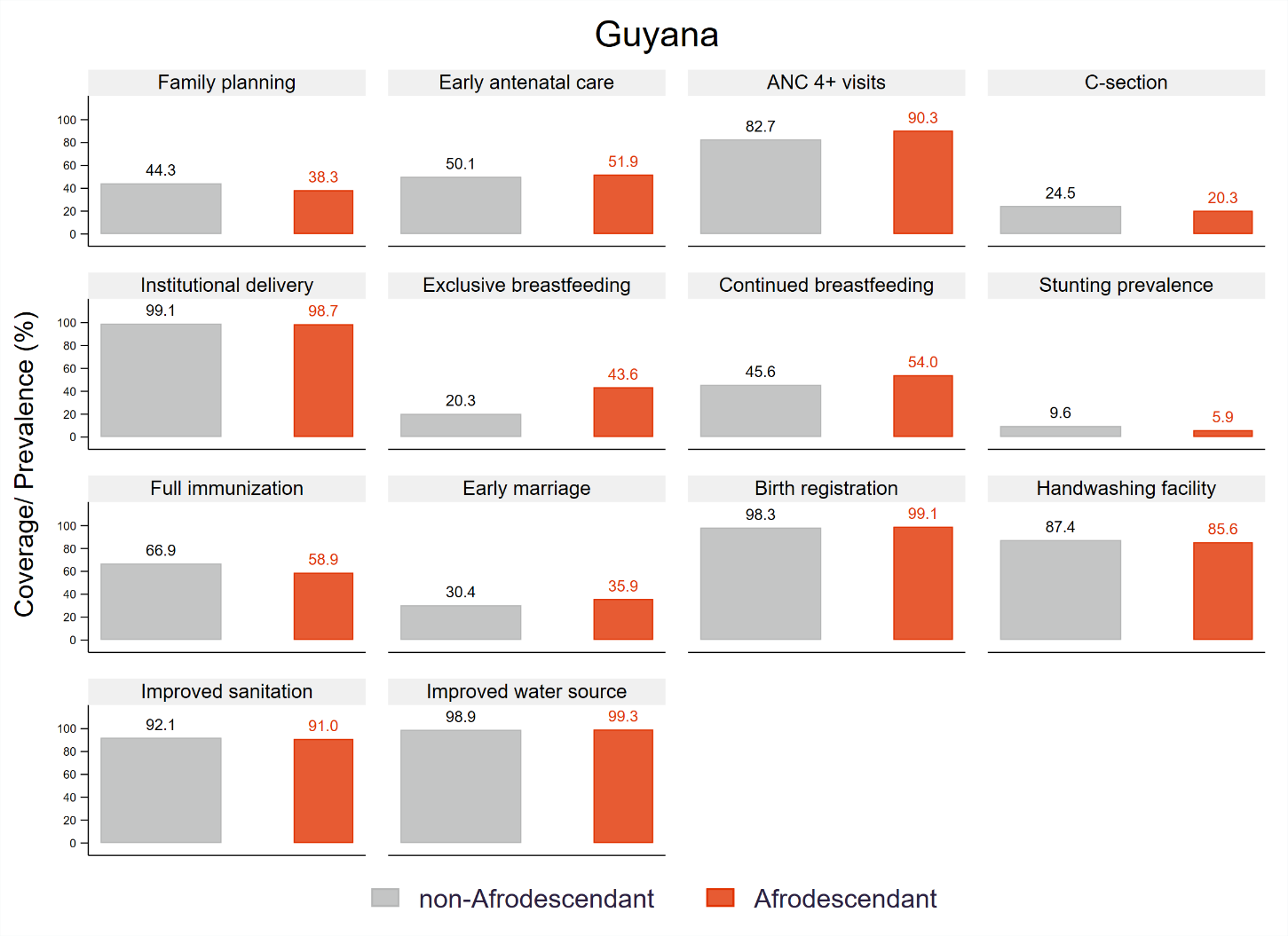

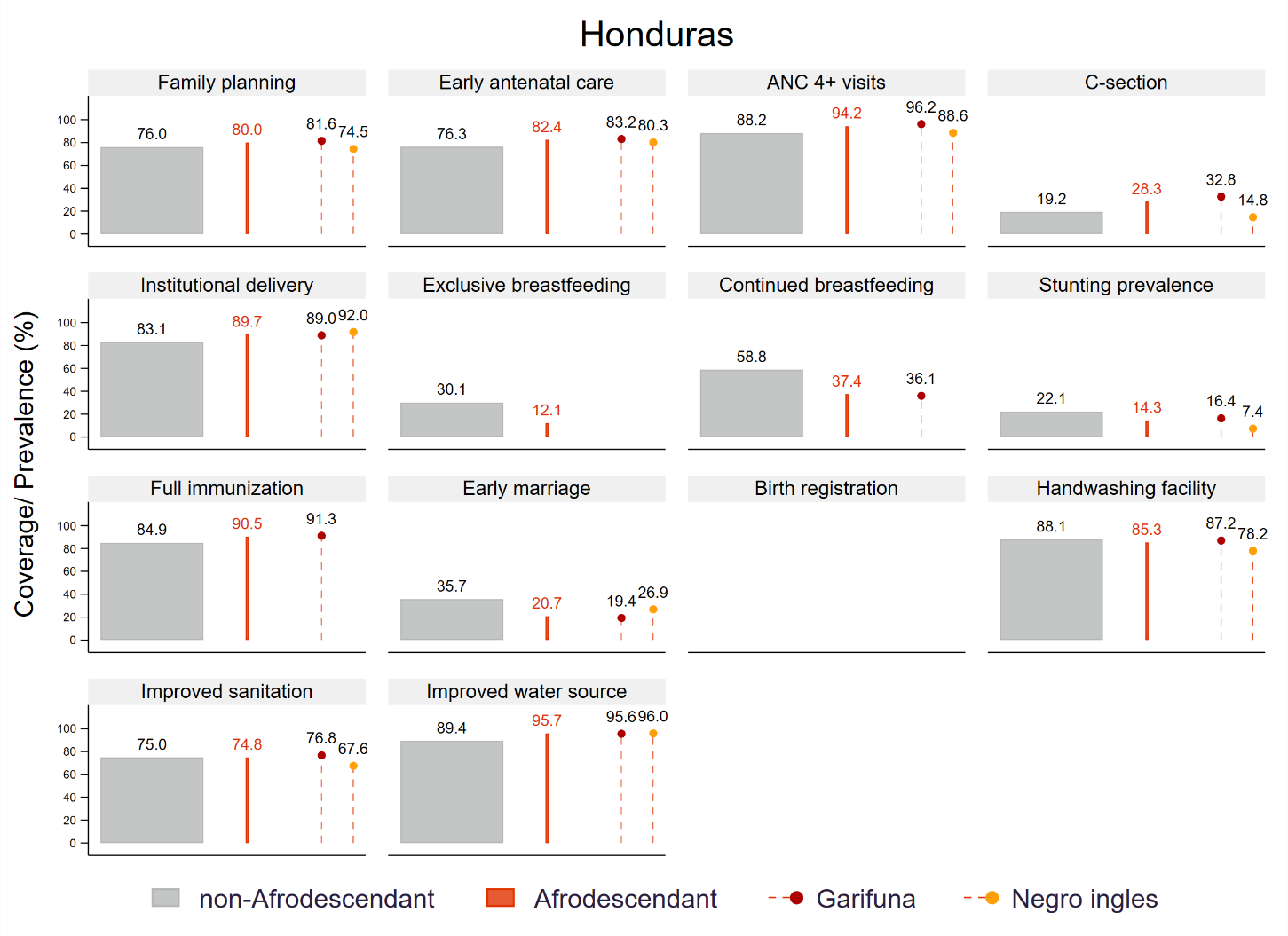

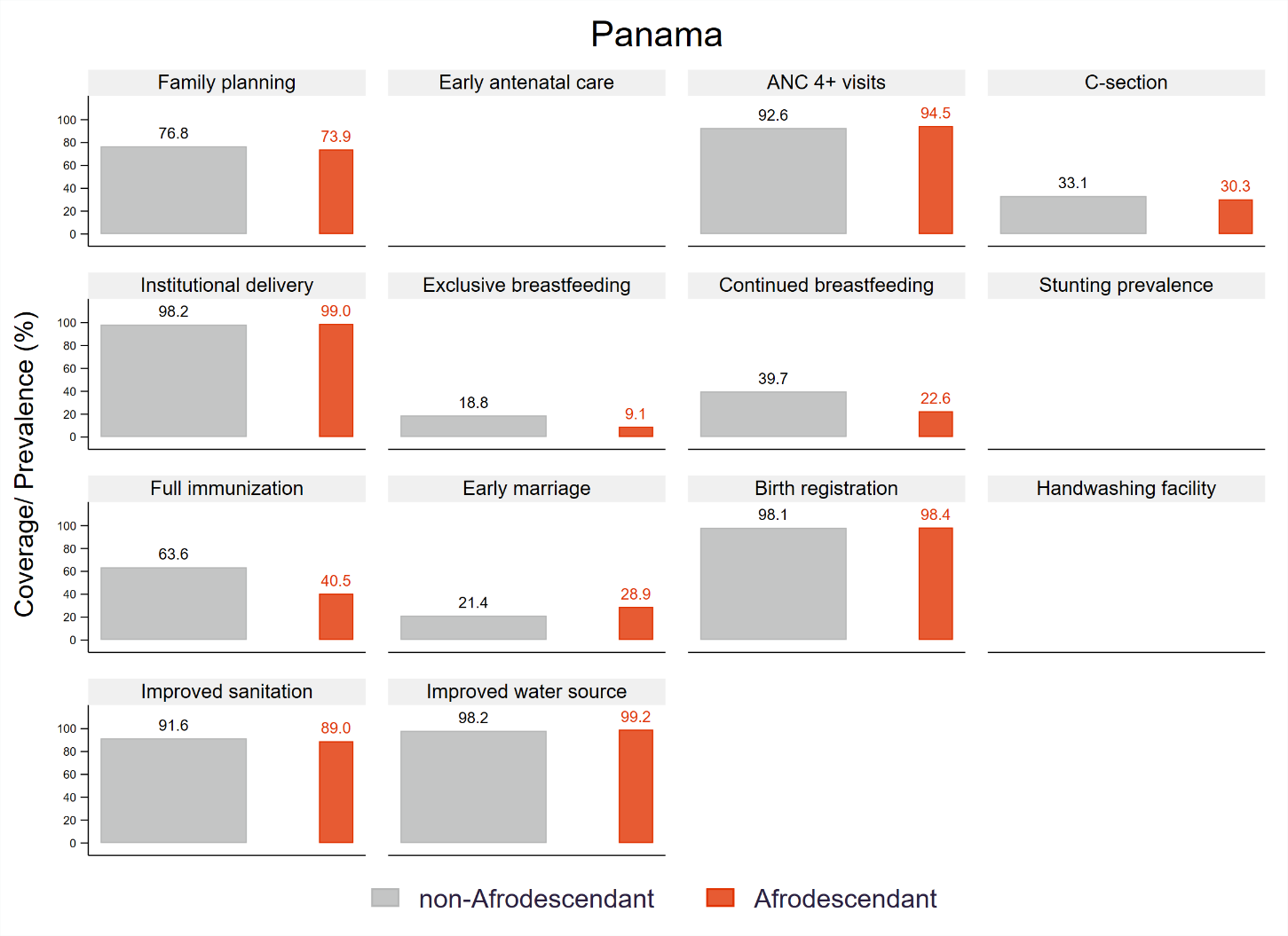

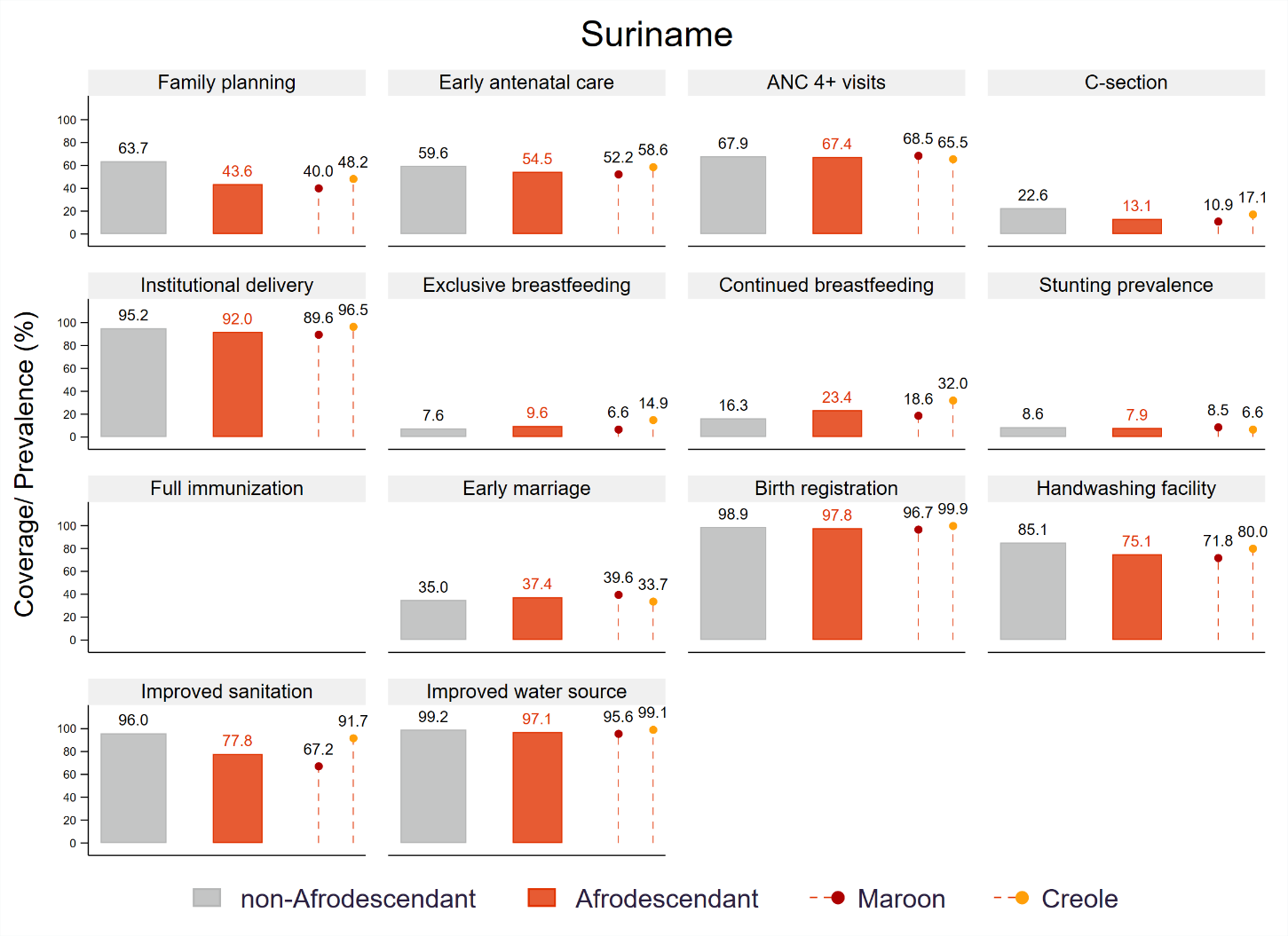

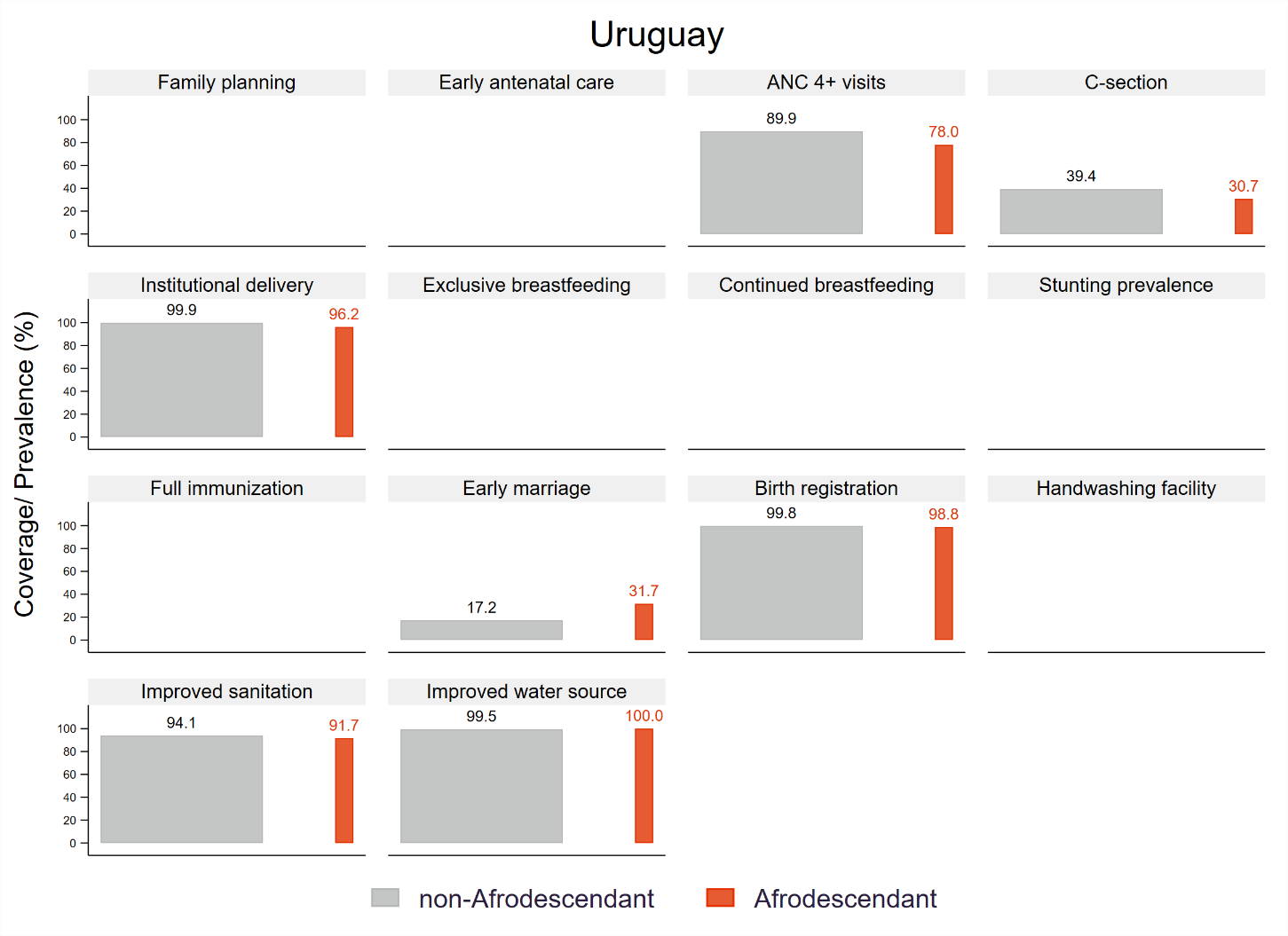


**Supplementary Figure S1 – Coverage or prevalence among Afrodescendant and non-Afrodescendant in Latin American and Caribbean countries.**

Bar thickness proportional to the group size. The lollipop bars indicate the estimates for Afrodescendant subgroups

**
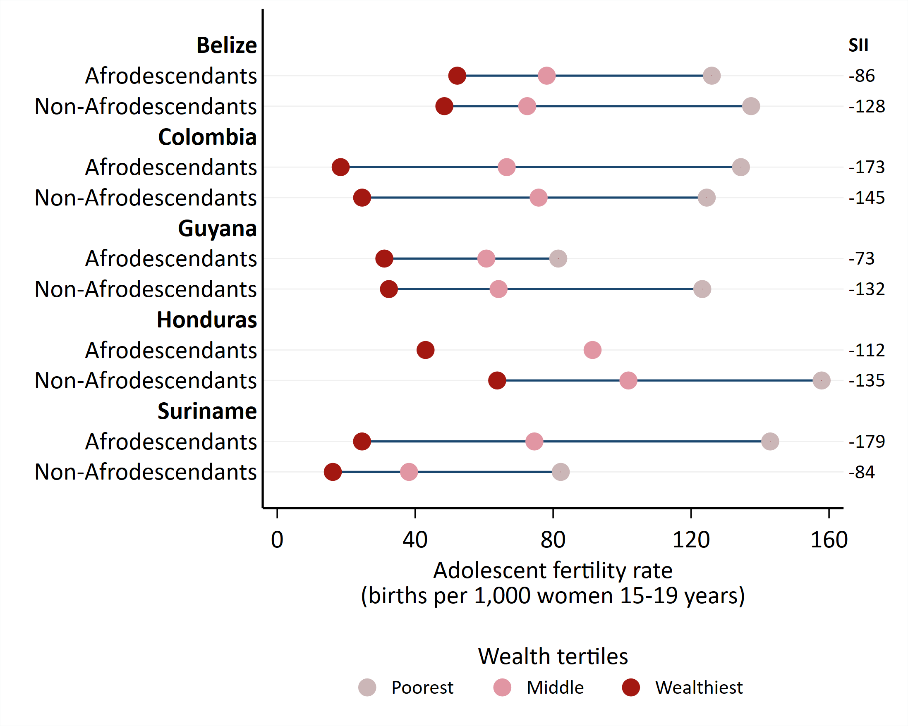
**

**
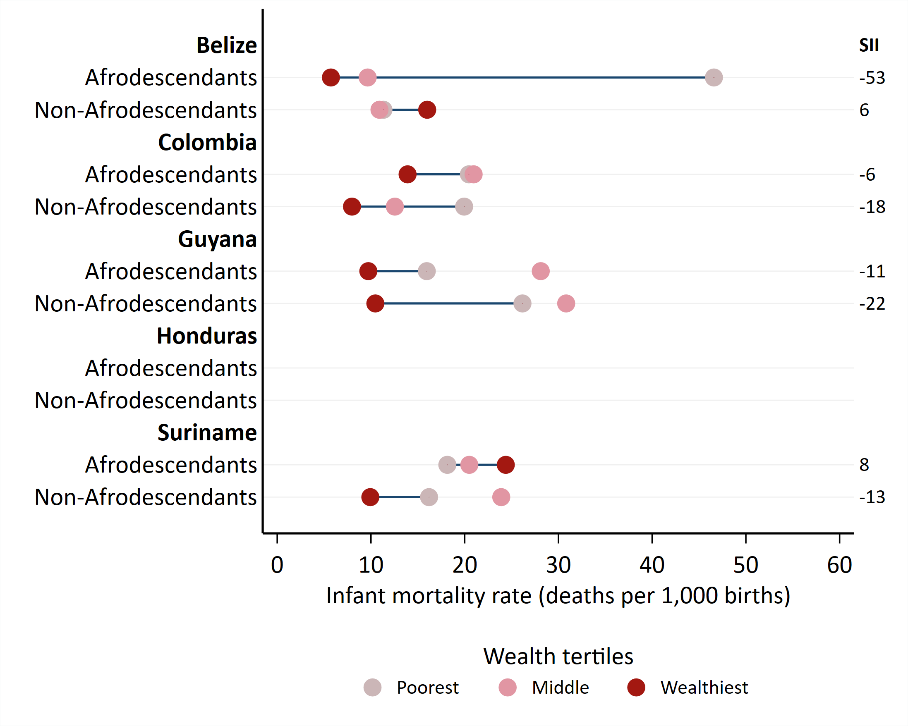

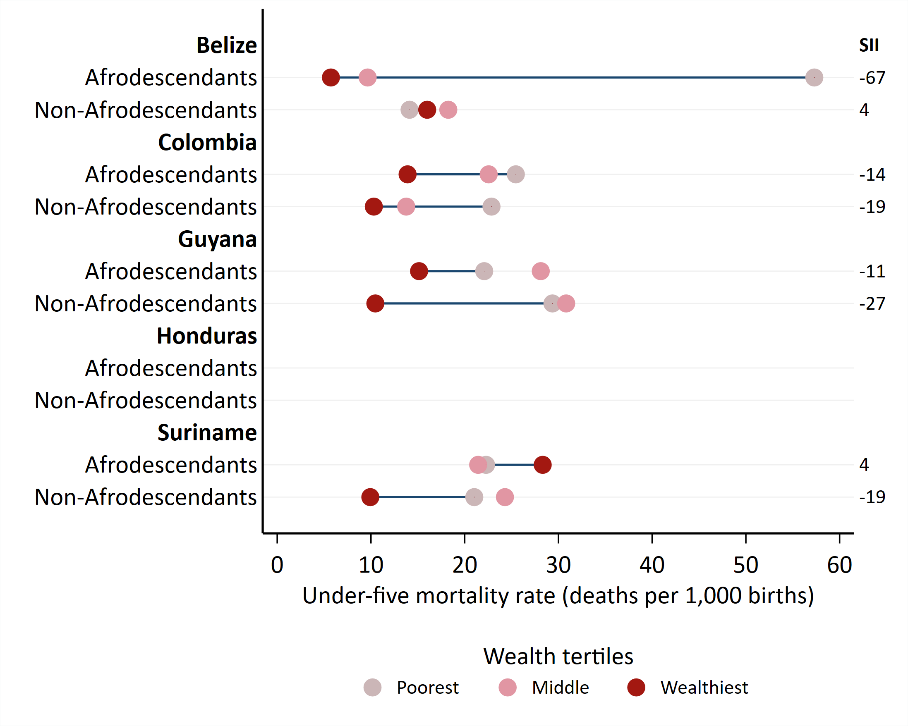
**

**Supplementary Figure S2 – Fertility and mortality rates by wealth tertiles for Afrodescendants and non-Afrodescendants, and the corresponding slope index of inequality.**


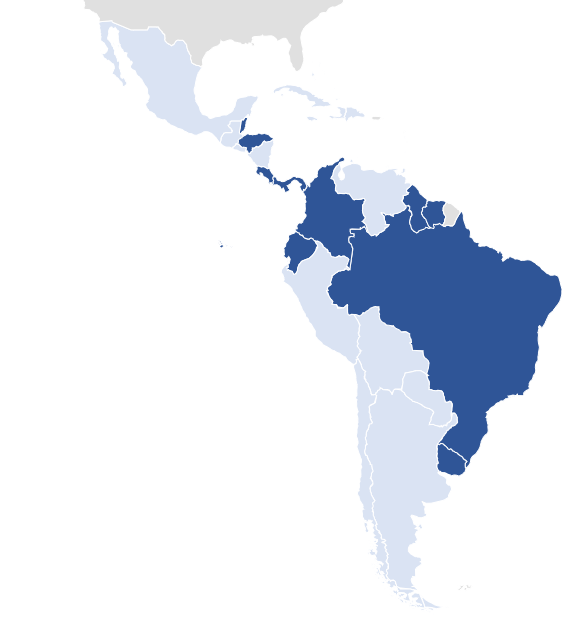


**Supplementary Figure S3 – Latin American and Caribbean countries (dark blue indicates those included in the analyses).**
